# Bortezomib exerts its anti-cancer activity through the regulation of Skp2/p53 axis in non-melanoma skin cancer cells and *C. elegans*

**DOI:** 10.1038/s41420-024-01992-7

**Published:** 2024-05-09

**Authors:** Kirti S. Prabhu, Fareed Ahmad, Shilpa Kuttikrishnan, Rari Leo, Tayyiba Akbar Ali, Mahmoud Izadi, Jericha M. Mateo, Majid Alam, Aamir Ahmad, Ammira S. Al-Shabeeb Akil, Ajaz A. Bhat, Joerg Buddenkotte, Ehsan Pourkarimi, Martin Steinhoff, Shahab Uddin

**Affiliations:** 1 Translational Research Institute, Academic Health System, Hamad Medical Corporation, Doha, 3050 Qatar; 2 Department of Dermatology and Venereology, Rumailah Hospital, Hamad Medical Corporation, Doha, 3050 Qatar; 3 Dermatology Institute, Academic Health System, Hamad Medical Corporation, Doha, 3050 Qatar; 4 Division of Genomics and Translational Medicine, College of Health and Life Sciences, Hamad Bin Khalifa University, Doha, 34110 Qatar; 5 Population Genetic and Genomics, Genetics and Metabolic Disorders Clinical Research Program, Precision Medicine of Diabetes Obesity and Cancer laboratory, Sidra Medicine, Doha, 26999 Qatar; 6 Department of Medicine, Weill Cornell Medicine-Qatar, Doha, 24144 Qatar; 7 College of Medicine, Qatar University, Doha, 2713 Qatar; 8 College of Health and Life Sciences, Hamad Bin Khalifa University, Doha, 34110 Qatar; 9 Department of Medicine, Weill Cornell Medicine, New York, NY 10065 USA; 10 Laboratory Animal Research Center, Qatar University, Doha, 2713 Qatar

**Keywords:** Skin cancer, Melanoma

## Abstract

Non-melanoma skin cancer (NMSC), encompassing basal and squamous cell carcinoma, is the most prevalent cancer in the United States. While surgical removal remains the conventional therapy with a 95% 5-year cure rate, there is a growing interest in exploring alternative treatment strategies. In this study, we investigated the role of Bortezomib (BTZ), a proteasome inhibitor, in NMSC. Using two NMSC cell lines (A431 and A388), we examined the effects of BTZ treatment. Our results demonstrated that 48 h of BTZ treatment led to downregulating Skp2 expression in both A431 and A388 cells while upregulating p53 expression, specifically in A388 cells. These alterations resulted in impaired cellular growth and caspase-dependent cell death. Silencing Skp2 in A388 cells with siRNA confirmed the upregulation of p53 as a direct target. Furthermore, BTZ treatment increased the Bax to Bcl-2 ratio, promoting mitochondrial permeability and the subsequent release of cytochrome C, thereby activating caspases. We also found that BTZ exerted its antitumor effects by generating reactive oxygen species (ROS), as blocking ROS production significantly reduced BTZ-induced apoptotic cell death. Interestingly, BTZ treatment induced autophagy, which is evident from the increased expression of microtubule-associated proteins nucleoporin p62 and LC-3A/B. In addition to cell lines, we assessed the impact of BTZ in an in vivo setting using *Caenorhabditis elegans* (*C. elegans*). Our findings demonstrated that BTZ induced germline apoptosis in worms even at low concentrations. Notably, this increased apoptosis was mediated through the activity of CEP-1, the worm’s counterpart to mammalian p53. In summary, our study elucidated the molecular mechanism underlying BTZ-induced apoptosis in NMSC cell lines and *C. elegans*. By targeting the skp2/p53 axis, inducing mitochondrial permeability, generating ROS, and promoting autophagy, BTZ demonstrates promising anti-cancer activity in NMSC. These findings provide novel insights into potential therapeutic strategies for controlling the unregulated growth of NMSC.

## Introduction

Non-melanoma skin cancer (NMSC) has experienced an alarming surge of 300 percent in the United States, making it the most prevalent form of cancer in the country [1]. The annual medical expenses associated with NMSC are estimated to reach $650 million [2]. The United Kingdom also faces a high incidence rate, with one in every thousand people diagnosed with NMSC annually, surpassing other European countries in its growth rate. The increasing prevalence of NMSC has elevated its status as a significant health concern in both nations [3, 4].

NMSC primarily consists of two subtypes: basal cell carcinoma (BCC) and squamous cell carcinoma (SCC). BCC, although the most common type of skin cancer, possesses the lowest potential for spreading and is considered less dangerous. On the other hand, SCC, which can arise from hair follicle stem cells, poses a more significant threat and accounts for 16% of all skin malignancies. While NMSC predominantly affects older people, with individuals over 60 representing nearly 80% of cases, there has been a notable rise in NMSC incidence among individuals younger than 35 [5–7]. These emerging trends underscore the urgency to advance our understanding of NMSC’s molecular pathology for improved identification, prevention, and treatment strategies [1].

Skp2, an oncoprotein and ubiquitin ligase, has been implicated in carcinogenesis and linked to poor prognosis in various human cancers, including oral squamous cell carcinoma [8–10]. Skp2 achieves its oncogenic effects through ubiquitination by degrading critical proteins, such as p21, p27, p57, E-cadherin, and FOXO1 [8, 11–14]. Interestingly, when inhibited, Skp2 has been shown to function as a tumor suppressor via Arf-p53-independent cellular senescence [15]. Recent research has unveiled that acetylation of Skp2 by p300 acetyltransferase leads to cytoplasmic retention, increasing cell migration by degrading E-cadherin [14].

BTZ (BTZ), a proteasome inhibitor, has shown beneficial effects in terms of survival improvement, tumor apoptosis, growth inhibition, and the suppression of angiogenesis and metastasis in preclinical in vivo studies. By suppressing the proteasome, BTZ stabilizes cyclin-dependent kinase inhibitors (p21 and p27), tumor suppressor p53, and proapoptotic proteins (Bid and Bax), thereby hindering cell cycle progression and inducing apoptosis [16–20]. Interestingly, the sensitivity to BTZ-induced apoptosis and chemosensitization appears to be cell-type dependent, with varying degrees of response observed with the p53 status [21–25].

To gain further insights, the model organism *Caenorhabditis elegans* (*C. elegans*) was employed to study the genetic basis of apoptotic cell death. *C. elegans* provides a valuable in vivo system for investigating drug effects and understanding their mechanisms due to the evolutionary conservation of apoptosis among multicellular organisms. In this study, we utilized genetic and high-resolution microscopy techniques to examine the impact of BTZ on *C. elegans* cell death. *Caenorhabditis elegans* (*C. elegans*) has been a robust system for studying the genetic background of various types of apoptotic cell death [26, 27]. Adult *C. elegans* contains two independent U-shaped germlines whose cells are at various stages of mitosis and the meiotic cell cycle. At the distal end of each germline are mitotically active cells, and as they progress proximally, they enter meiosis I. Multiple mechanisms, such as physiological and DNA damage-induced germ cell apoptosis, can induce apoptosis in the proliferative germline of adult worms [28]. However, only the meiotic germ cells at the late pachytene stage can undergo apoptosis. Despite different apoptotic pathways, *C. elegans* requires core apoptotic machinery to induce cell death of most cells, consisting of EGL-1, BCL-2-like protein CED-9, CED-4, a mammalian APAF-1 orthologue, and a caspase CED-3 [29–33]. Since apoptosis is evolutionarily conserved among multicellular organisms, *C. elegans* provides a unique in vivo model system to screen drugs and comprehend their mechanism. In this study, we have used genetic and high-resolution microscopy to characterize the effect of BTZ on *C. elegans* cell death. Our findings revealed that BTZ induced germline apoptosis in worms even at low concentrations. Notably, this increased apoptosis was mediated through the activity of CEP-1, the worm’s counterpart to mammalian p53. These results highlight the conserved nature of apoptosis and the relevance of *C. elegans* as a model system for studying drug effects and unraveling underlying mechanisms. The observations made in *C. elegans* provide crucial evidence supporting the potential of BTZ as an effective therapeutic agent in NMSC and other malignancies where apoptosis plays a vital role.

The present work aimed to elucidate the physiological role of BTZ in NMSC cancer progression by exploring its effects on cell proliferation, apoptosis, cell cycle regulation, migration, and invasion. Our findings demonstrated that BTZ effectively suppressed cell proliferation, induced apoptosis, arrested cell division in the G0/G1 phase, and inhibited migration and invasion. Additionally, we uncovered that BTZ exerts its anti-cancer effects in NMSC by downregulating the Skp2 pathway and upregulating the p53 protein. Collectively, our study highlights the potential of BTZ as a promising therapeutic agent for treating NMSC.

Combining our findings in NMSC cell lines and the *C. elegans* model adds further depth and significance to our understanding of the molecular mechanisms underlying BTZ-induced apoptosis. This comprehensive approach enhances the translational potential of our research, potentially paving the way for future preclinical and clinical investigations targeting NMSC and other related cancers.

## Results

### BTZ inhibits the growth of NMSC cells, induces DNA damage response, and promotes cell death

**Fig. 1:**
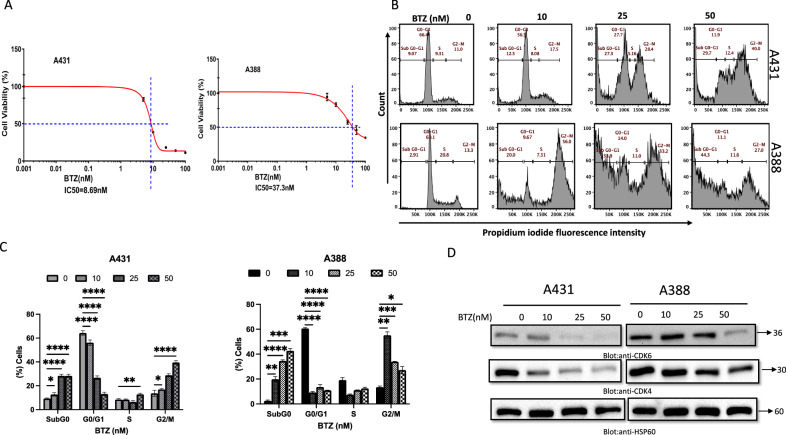
Effects of Bortezomib (BTZ)–on cell proliferation and cell cycle. **A** BTZ inhibits the viability of A431 and A388 in a concentration-dependent after treatment with BTZ for 48 h. Cell cycle fraction analysis and cell cycle regulating proteins in response to BTZ. Upon treatment with BTZ as indicated, NMSC cells were analyzed by flow cytometry wherein BTZ significantly enhanced SubG0 fraction in A431 and A388 (**B**, **C**) and by (**D**) western blot analysis where expression of CDK4, CDK6, and HSP60 was examined. The graph displays the mean ± SD of three independent experiments. *P < 0.05, **P < 0.01, ***P < 0.001. The original western blots and quantification graphs can be found in Supplementary Files 1 and 2, respectively.

**Fig. 2:**
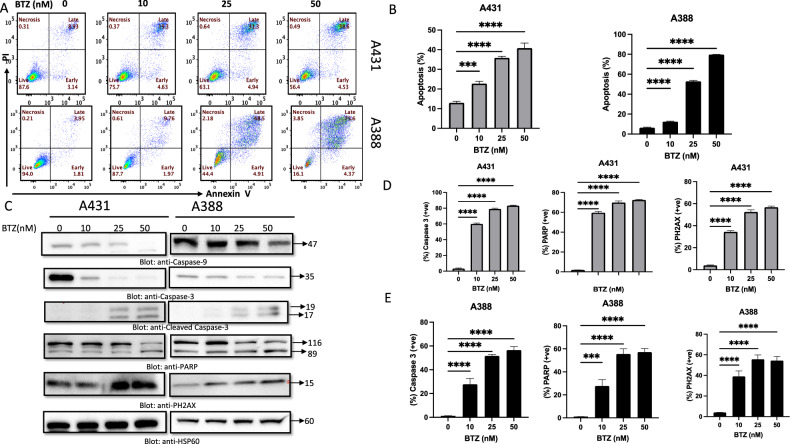
BTZ induces apoptosis in NMSC cells. **A**, **B** A431 and A388 cells were treated with BTZ in a dose-dependent manner and analyzed by flow cytometry for apoptosis. BTZ- mediated caspase cascade activation followed by DNA double-strand breakage in A431 and A388 cells as analyzed by **C** western blot and **D**, **E** flow cytometry. The original western blots and quantification graphs can be found in Supplementary Files 1 and 2, respectively.

**Fig. 3:**
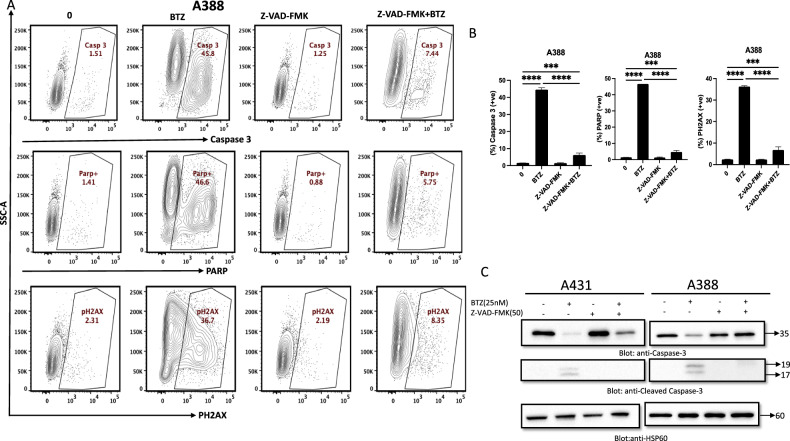
z-VAD-FMK reversed BTZ-induced caspase activation in NMSC cells. NMSC cells A431 and A388 were treated with BTZ and z-VAD-FMK alone and in combination for 48 h. Caspase activation induced by BTZ in A431 and A388 cells was reversed by z-VAD-FMK as analyzed by flow cytometry (**A**, **B**) and western blot (**C**). The graph displays the mean ± SD of three independent experiments. *P < 0.05, **P < 0.01, ***P < 0.001. The original western blots and quantification graphs can be found in Supplementary Files 1 and 2, respectively.

Our investigation focused on the effect of BTZ on NMSC cell lines A431 and A388. The viability and proliferation of these cells, treated with varying concentrations of BTZ for 48 h, were assessed using the CCK-8 assay. BTZ considerably impaired cell viability, with the necessary concentration to achieve this effect ranging from 10–50 nM, as indicated by IC_50_ values (A431-8.69 nM and A388-37.3 nM) (Fig. 1A). To further validate the cytotoxic effect of BTZ on NMSC cell lines, a live/dead assay, using calcein and ethidium bromide (EtBr) homodimer fluorescent probes, was utilized. Results demonstrated an increasing trend of dead cells and a concomitant decline in live cells following BTZ treatment, providing compelling evidence for BTZ’s cytotoxicity (Supplementary Fig. 1A). Moreover, our study explored the apoptosis-inducing potential of BTZ. Notably, BTZ exposure increased cell fraction in SubG0/G1 phase, implying possible cell cycle arrest (Fig. 1B, C). This, coupled with the observed suppression of cyclin-dependent kinases (CDKs) CDK4 and CDK6 expression (Fig. 1D), hints at the crucial role of CDKs in BTZ’s anti-cancer effects. Distinct apoptotic morphological alterations, such as cell rounding and shrinkage, were observed in BTZ-treated cells (Supplementary Figure 1B). The Annexin V/PI dual staining and the elevated cleaved isoforms of caspase-9 and caspase-3 alongside increased PARP cleavage further confirmed these observations (Fig. 2A–C). Consistent with these findings, flow cytometry revealed a surge in caspase-3 and PARP cleavage activities upon BTZ treatment (Fig. 2D, E, Supplementary Figures 2A, B). To unequivocally confirm caspase activation’s role in BTZ-mediated apoptosis, cells were pretreated with pan-caspase inhibitor z-VAD-FMK, resulting in the reversal of the BTZ-induced apoptotic effects. In addition, we observed an induction of DNA damage following BTZ treatment, as indicated by the enhanced phosphorylation of H2AX, a marker for DNA double-strand breaks (Fig. 3A–C). Finally, the specificity of BTZ’s effects was confirmed by demonstrating the lack of its impact on the levels of Skp2 and PARP in normal human epidermal keratinocytes treated similarly to cancerous cells (Supplementary Fig. 3A). These findings collectively highlight BTZ’s potential as a targeted therapeutic agent for NMSC.

### BTZ suppresses proteasome activity in NMSC cells

**Fig. 4:**
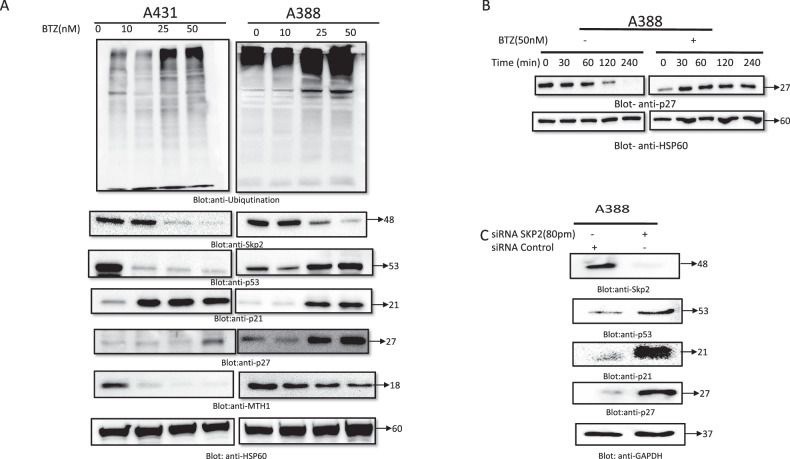
BTZ suppresses proteasome activity in NMSC cells. **A** A431 and A388 cells were treated with different doses of BTZ for 48 h as indicated. After cell lysis, equal amounts of proteins were separated by SDS-PAGE, transferred to the PVDF membrane, and immunoblotted with antibodies of anti-ubiquitin, Skp2, p53, p27, p21, MTH1 and HSP60. **B** BTZ treatment of A388 cells causes the stabilization of p27. A388 cells were treated with and without 50 nM of BTZ for 48 h. Cells were then treated with 10 μM Cyclohexamide for 30, 60, 120 and 240 min. Cells were lysed and equal amounts of proteins were separated by SDS-PAGE, transferred to PVDF membrane, and immunoblotted with antibodies against p27and HSP60 as indicated. **C** SKP2 siRNA expression downregulates SKP2 and upregulated p53, p27, p21. A388 cells were transfected with Scrambled siRNA (100 nm) and SKP2 siRNA (50–100 nm) using Lipofectamine 2000 as described in the “Methods” section. After 48 h of transfection, cells were lysed and equal amounts of proteins were separated by SDS-PAGE, transferred to the PVDF membrane, and immunoblotted with antibodies against Skp2,p53, p27, p21, and GAPDH as indicated. The original western blots and quantification graphs can be found in Supplementary Files 1 and 2, respectively.

P300 and p53 are recognized as crucial in the cellular response to oncogenic stress, primarily through their roles in tumor suppression via cell cycle arrest and apoptosis induction. The stabilization and transcriptional activity of p53 depends upon p300-mediated acetylation of p53 [34]. It has been reported that Skp2 interacts with p300 via the CH1 and CH3 domains of p300, forming a complex. These domains are key to the interaction between p300 and p53. However, an ectopic expression of Skp2 leads to insufficient p300-mediated p53 acetylation and transcriptional activity, thus hindering p53’s interactions with p300 [35]. Skp2’s overexpression exhibits an oncogenic property that shields cancer cells from apoptosis, even in the face of DNA damage [35, 36]. This intriguing interplay led us to speculate about the potential impact of BTZ on the expression of Skp2 and p53 and their potential role in restraining cancer cell growth. Furthermore, studies have demonstrated that Skp2, an oncogene, plays a pivotal role in regulating the SCF protein complex, which is implicated in the ubiquitination and proteasome-mediated degradation of p27 [37]. Given this context, we aimed to explore whether BTZ might target these proteins and consequently modulate the expression of downstream proteins such as Skp2, p53, p21, and p27. Indeed, our results clearly indicated that BTZ downregulates Skp2 expression, thus promoting the expression of p53 (in A388), p21, and p27 in both A431 and A388 cell lines. The impact of BTZ’s proteasome inhibition on A431 and A388 cells after a 48-h exposure was demonstrated by the accumulation of polyubiquitinated proteins. MTH1 (MutT Homolog 1) plays a critical role in cellular protection against oxidative stress by cleaning the nucleotide pool. It hydrolyzes oxidized purine nucleoside triphosphates, preventing their incorporation into DNA and averting potential mutagenesis. This enzymatic function is particularly crucial in cancer, where oxidative stress levels are markedly elevated [38]. Within our study, we explored the regulatory mechanisms that modulate MTH1 expression in skin cancer cell lines. Our investigation revealed that Skp2, a component of the SCF (Skp1-Cul1-F-box protein) E3 ubiquitin ligase complex, is a key player in this regulation. We demonstrated that Skp2 enhances MTH1 stability through K63-linked polyubiquitination. This post-translational modification does not target MTH1 for degradation but rather stabilizes the protein, as evidenced by our observations that silencing Skp2 diminishes while overexpressing Skp2 enhances the K63-linked polyubiquitination and subsequent stabilization of MTH1. Furthermore, our data on the treatment with Bortezomib (BTZ), a proteasome inhibitor, show a decrease in MTH1 levels in A431 and A388 skin cancer cell lines, correlating with the downregulation of Skp2 (Fig. 4A). This finding adds a layer of complexity to the understanding of MTH1 regulation in cancer, suggesting a potential therapeutic angle by targeting the Skp2-MTH1 axis.

Furthermore, to discern the effect of BTZ on p27 stability, a cycloheximide chase assay was employed. BTZ treatment resulted in p27 accumulation, signifying that BTZ’s upregulation of p27 can be attributed to increased p27 stability (Fig. 4B). Additionally, our experiment involving Skp2-specific siRNA/shRNA to silence Skp2 expression in A388 cells led to a noteworthy upregulation of p21 and p27 levels. Interestingly, this silencing also caused an increase in p53 levels, underscoring a robust relationship between Skp2 downregulation and the activation of the tumor suppressor gene p53 (Fig. 4C). These findings collectively underscore BTZ’s potential to regulate key proteins involved in cell cycle control and apoptosis, offering valuable insights for cancer therapeutics.

### BTZ treatment activates caspase-8 and downregulates Bcl-2 proteins in NMSC cells

**Fig. 5:**
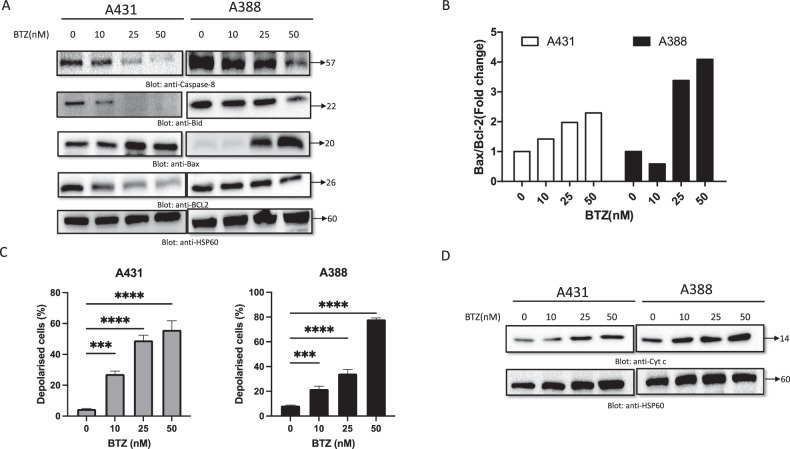
BTZ-induced mitochondrial signaling pathways in NMSC cells. BTZ treatment causes alteration in Bcl-2 expression. **A** A431 and A388 cells were treated with increasing doses of BTZ for 48 h, as indicated. After cell lysis, equal amounts of proteins were separated by SDS-PAGE, transferred to the PVDF membrane, and immunoblotted with antibodies against caspase-8, Bid, Bax, Bcl-2, and HSP60. **B** Data obtained from immunoblot analysis of Bax and Bcl-2 in A431 and A388 were used to evaluate the effects of BTZ on Bax/Bcl-2 ratio. Densitometric analysis of Bax and Bcl-2 bands was performed using AlphaImager Software (San Leandro, CA, USA), and data (relative density normalized to HSP60) were plotted as Bax/Bcl-2 ratio. Treatment with BTZ caused loss of mitochondrial membrane potential in NMSC cells. **C** A431 and A388 cells were treated with increasing concentrations of BTZ for 48 h and analyzed by flow cytometry. The graph displays the mean ± SD of three independent experiments (*P < 0.05, **P < 0.01, and ***P < 0.001). BTZ-induced the release of cytochrome c. **D** A431 and A388 cells were treated in the presence and absence of BTZ for 48 h. Cytoplasmic fraction was isolated as described in the methodology. Cell extracts were separated on SDS-PAGE, transferred to the PVDF membrane, and immunoblotted with an antibody against cytochrome c and HSP60. The original western blots and quantification graphs can be found in Supplementary Files 1 and 2, respectively.

To explore the role of caspase-8 in apoptosis [39], we treated A431 and A388 cells with BTZ for a period of 48 h. We observed an activation of caspase-8 upon BTZ treatment, which initiated the cleavage of the Bid. This cleaved Bid was then translocated to the mitochondria, influencing the regulation of Bcl-2 family members, specifically Bax and Bcl-2. While Bax, a proapoptotic molecule, promotes apoptosis, Bcl-2, an anti-apoptotic molecule, counteracts it, thereby creating a delicate balance that can shift to cell resistance against chemotherapy and radiation if disturbed [40]. During the 48-h BTZ treatment, both A431 and A388 cell lines demonstrated a dose-dependent decrease in Bcl-2 and an increase in Bax expression (Fig. 5A). A densitometric analysis confirmed this finding by revealing an elevated Bax/Bcl-2 ratio (Fig. 5B), which aligns with the involvement of a mitochondnurial-mediated pathway in the apoptosis process. We employed flow cytometry to examine the ability of BTZ to induce mitochondrial membrane depolarization. After 48 h of BTZ treatment, both NMSC cell lines exhibited a reduction in mitochondrial membrane potential (Fig. 5C). This observation suggests BTZ’s role in disrupting mitochondrial functionality, triggering the apoptotic cascade. The release of cytochrome c is a crucial event within the apoptotic cascade, typically triggered by damage to the mitochondrial membrane. After 48 h of BTZ treatment, we analyzed mitochondrial cytochrome c release via western blotting in A431 and A388 cells. A clear upregulation of cytochrome c in the cytosolic fraction was observed after BTZ treatment (Fig. 5D), exhibiting a dose-dependent effect. The release of cytochrome c is a recognized step to initiate the caspase cascade activation, a key hallmark of programmed cell death [41, 42].

In summary, our findings suggest that BTZ’s potential anti-cancer efficacy could be largely attributed to its influence on the caspase-8-Bid-Bax/Bcl-2 mitochondrial pathway. This pathway is critical in the mediation of apoptotic cell death, and its modulation by BTZ opens new possibilities for targeting resistant cell lines in cancer treatment.

### Impact of BTZ on reactive oxygen species production and apoptosis induction

**Fig. 6:**
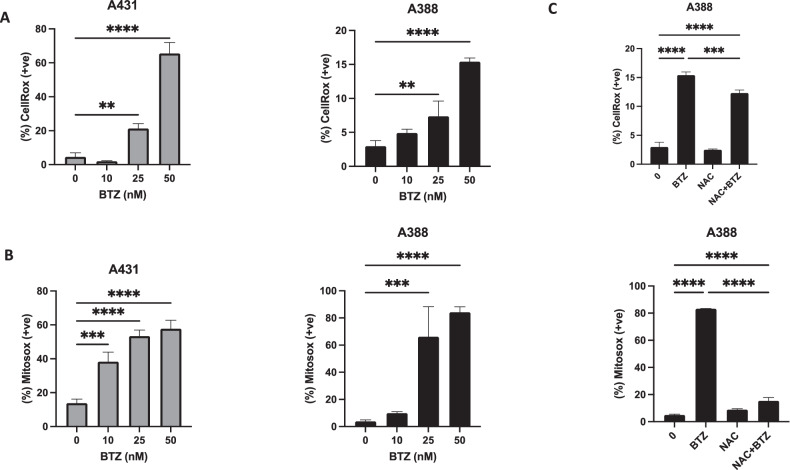
BTZ -mediated reactive oxygen species (ROS) generation in NMSC cells. A431 and A388 were treated with BTZ for 48 h. **A**, **B** Cellrox and mitoSOX assays were performed to evaluate the level of ROS by flow cytometry as described in the methodology. NAC-pretreated NMSC cells prevented BTZ-mediated activation of ROS. **C** A388 cells were pretreated with 10 mM NAC and then subsequently treated with 25 nM BTZ as indicated for 48 h. Th graph displays the mean ± SD (standard deviation) fold change release of ROS of three experiments (*P < 0.05, **P < 0.01, ***P < 0.001). The original western blots and quantification graphs can be found in Supplementary Files 1 and 2, respectively.

**Fig. 7:**
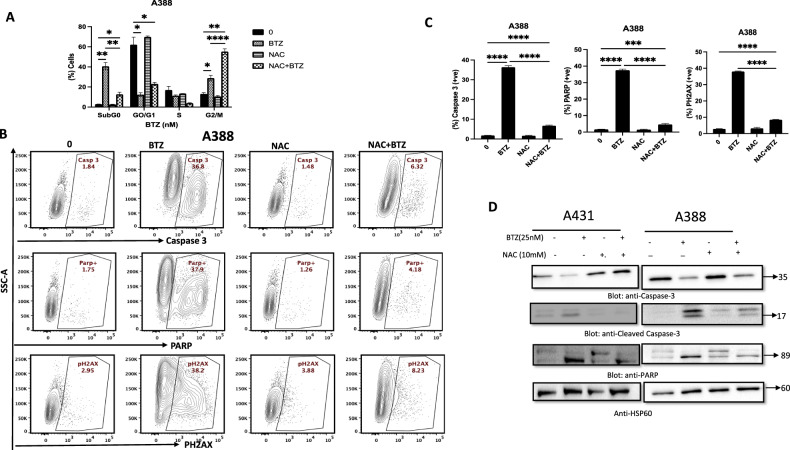
BTZ A-mediated ROS generation involved in apoptotic cell death in NMSC cells. N-acetylcysteine (NAC) pretreated leukemic cells abrogated the BTZ-induced increase in SubG0 fraction in (**A**) A388 cells were pretreated with 10 mM NAC followed by 25 nM BTZ for 48 h, and cell cycle fraction was measured by flow cytometry. NAC pretreated leukemic cells prevented BTZ-mediated activation of caspases. A388 cells were pretreated with 10 mM NAC, then subsequently treated with 25 nM BTZ as indicated for 48 h and subjected to flow cytometry (**B**, **C**) and (**D**) for western blot lysed cell extracts were separated on SDS-PAGE, transferred to PVDF membrane, and immunoblotted with an antibody against procaspase-3, cleaved caspase-3, PARP, and HSP60. The graph displays the mean ± SD of three independent experiments. *P < 0.05, **P < 0.01, ***P < 0.001. The original western blots and quantification graphs can be found as Supplementary Files 1 and 2 respectively.

Reactive oxygen species (ROS) play a pivotal role in normal cell proliferation and differentiation processes [43]. Disturbances in ROS levels can trigger a cascade of events leading to apoptosis, including loss of mitochondrial membrane potential, the release of apoptosis-inducing substances, activation of caspases, and nuclear condensation [40, 44]. To assess the effect of BTZ on ROS levels, we exposed A431 and A388 cells to increasing doses of BTZ for 48 h, followed by ROS quantification. Both cell lines exhibited a substantial, dose-dependent elevation in cellular and mitochondrial ROS production (Fig. 6A, B). As an additional step, we employed the ROS scavenger N-acetyl-L-cysteine (NAC) to counteract the BTZ-induced ROS production. Cells pretreated with NAC effectively abrogated BTZ-triggered ROS generation, hinting at a potential strategy to mitigate the oxidative stress induced by BTZ treatment (Fig. 6C). To further ascertain the role of ROS in BTZ-induced apoptosis, we examined the impact of NAC on BTZ-mediated cell death. We observed that NAC efficiently reversed the BTZ-induced increase in the SubG0/G1 fraction (Fig. 7A). Furthermore, NAC pre-treatment inhibited BTZ-induced caspase cascade activation and PARP cleavage (Fig. 7B–D). This collective evidence strongly suggests that BTZ-induced apoptosis is mediated via a ROS-dependent mechanism.

### BTZ induces apoptosis in *C. elegans*

To analyze the effect of BTZ on *C. elegans*, we used cytological markers and DIC optics-based fluorescent microscopy. *C. elegans* has been instrumental in understanding apoptosis mechanism and degradation of apoptotic cells [45, 46]. During *C. elegans* development and as part of the worm’s normal developmental program, 131 somatic cells are eliminated by developmental apoptosis. Also, in adult worms, nearly 50% of the germ cells undergo physiological cell death, indispensable for preserving tissue homeostasis. The physiological cell death that occurs independently of external stimuli is restricted to the late pachytene-staged meiotic germ cells [47]. In addition to developmental and physiological cell death, genotoxic insult induces DNA damage-induced apoptosis in the germline of worms, mediated by CEP-1, the *C. elegans* orthologue of the mammalian p53. Regardless of the apoptotic death modality, dying cells must be engulfed and degraded by the neighboring cells. The engulfment of cells undergoing apoptosis is mediated by phagocytosis that involves an evolutionary conserved transmembrane receptor protein encoded by the *ced-1* gene, the orthologue of mammalian Scavenger Receptor from Endothelial Cells or SREC. Therefore, using CED-1 tagged GFP enables the detection of apoptotic cell death and their engulfment by the neighboring cells [48].

**Fig. 8:**
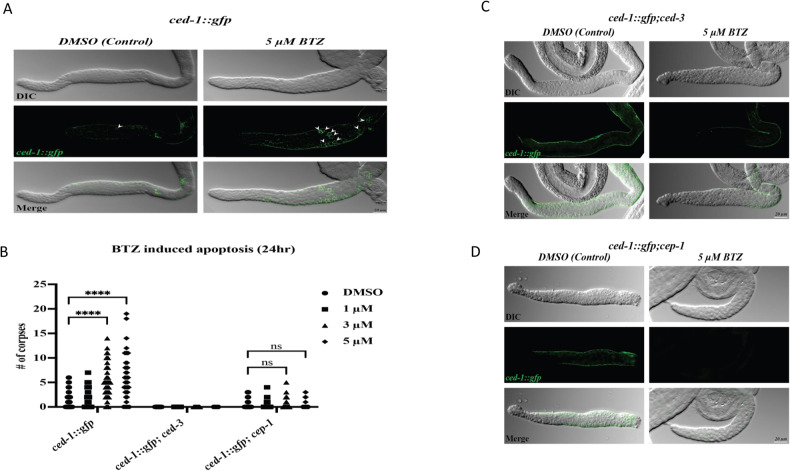
BTZ induces apoptosis in *C. elegans*. **A** Apoptotic corpses in *ced-1::gfp* 24 h after treatment with 5 µM BTZ (BTZ). Increased apoptotic corpses were detected in the extracted germlines of *ced-1::gfp* worms upon treatment with BTZ. There are 1–2 corpses in control (DMSO treated) germlines, while upon 5 µm BTZ, 8–10 corpses can be observed by engulfment marker CED-1, fused with GFP. **B** The graph shows a significantly increased number of apoptotic corpses in the germlines of worms treated with various dosages of BTZ in ced-1::gfp strain (*n* = 45). The caspase-null strain (*ced-3* mutants) confirmed the apoptosis, where no apoptotic corpses were observed in *ced-1::gfp;ced-3* (*n* = 40). *cep-1/p53* null mutant was used to confirm the BTZ-induced apoptosis via the DNA damage pathway. The graph shows the basal level of apoptosis in *ced-1::gfp;cep-1* strain among treated versus control worms, showcasing its dependency on *cep-1*/p53 (*n* = 45). Arrows mark the apoptotic corpses in the pachytene region of the germlines. DIC: Differential Interference Contrast. GFP: Green Fluorescent Protein. (****P < 0.0001). Genetic characterization of BTZ-induced apoptosis. **C** Comparing *ced-1::gfp;ced-3* worms treated with BTZ (BTZ) versus control showed no significant differences in the number of apoptotic corpses as the apoptosis is not executed in the absence of CED-3 in *ced-3* null mutant, indicating caspase dependency of BTZ-induced apoptosis. **D** In the *cep-1/*p53 null mutant (*ced-1::gfp;cep-1*), apoptosis is not detected, suggesting the DNA damage-induced apoptosis upon BTZ treatment. All GFP signal intensities were normalized to their corresponding control. DIC differential interference contrast, GFP green fluorescent protein.

To test if the BTZ induces apoptosis in *C. elegans*, we treated wild-type worms expressing *ced-1::gfp* with different concentrations of BTZ. Expression and cluster formation of CED-1::GFP around the dying cells is used as a cytological marker to detect apoptosis [48]. In line with our in vitro data, BTZ strongly induces apoptotic death in the pachytene zone of the *C. elegans* germline upon 24 h of BTZ exposure with various concentrations (Fig. 8A). To quantify the number of apoptotic corpses, we counted the number of CED-1::GFP-positive cells with or without BTZ treatment and observed a significant increase in apoptotic cells upon BTZ exposure (Fig. 8B).

In *C. elegans*, CED-3 caspase is the most downstream component of the core apoptotic machinery and is required to execute various types of apoptotic death [29, 32]. To further confirm that BTZ-induced apoptosis and the observed CED-1::GFP accumulation depend on the apoptosis executing caspase, we analyzed the effect of BTZ on the caspase-null mutant background. Upon treating ced-1::gfp;cep-1(gk138) worms with BTZ, we could only detect the average level of physiological apoptotic cells in both control and drug-treated worms, and the majority of the ced-1::gfp;cep-1(gk138) treated worms showed no GFP-positive germ cells caspase, confirming that BTZ induces caspase-dependent apoptotic death (Fig. 8B, C).

### BTZ induces *p53*/*cep-*1 dependent apoptosis in *C. elegans*

In the proliferative germline of *C. elegans*, germ cells can undergo apoptosis either by physiological cues or in response to DNA-damaging agents. DNA damage-induced apoptosis requires CEP-1, the *C. elegans* counterpart of the mammalian p53 [49]. Therefore, to characterize whether BTZ-induced apoptosis is through p53 activity, we treated the *ced-1::gfp;cep-1 (Ig12501)* with BTZ. *cep-1 (Ig12501)* is a null mutant that will not induce apoptosis when worms are exposed to genotoxic insults. Upon treating *ced-1::gfp;cep-1(gk138)* worms with BTZ, we could only detect the normal level of physiological apoptotic cells in both control and drug-treated worms, and the majority of the *ced-1::gfp;cep-1(gk138)* treated worms showed no GFP-positive germ cells. This observation strongly suggests that increased apoptosis cell death imposed by BTZ depends on the function of CEP-1/ p53 (Fig. 8B, D). Our data revealed that apoptosis induced by BTZ is through a DNA-damage pathway by activating *p53/cep-1*, which subsequently activates the core apoptotic machinery.

### BTZ-induced alterations in the expression of cell migration-associated proteins

Cancer development and progression hinge on four key traits: cellular migration, invasion, metastasis, and angiogenesis, each intricately linked to the tumor microenvironment [50]. An essential component of these processes is Matrix Metalloproteinases (MMPs), enzymes known to modify cell-cell and cell-extracellular matrix (ECM) interactions [51]. They achieve this by degrading various cell adhesion molecules, consequently influencing tumor behavior. Existing research increasingly points towards diverse roles of MMPs and their natural antagonists - Tissue Inhibitors of Metalloproteinases (TIMPs) - throughout cancer evolution, with differential expression levels seen in different cancer stages [52]. Given this context, our study sought to explore the effect of BTZ on the expression of specific MMPs - MMP-2 and MMP-9 - and TIMP-1, an inhibitory regulator of MMPs. Utilizing western blotting analysis, we discerned notable changes in the expression levels of these proteins when exposed to BTZ at concentrations of 25 and 50 nM. Our findings revealed increased TIMP-1 expression, in tandem with a decrease in MMP-2 and MMP-9 protein levels (refer to Supplementary Fig. 3B). This suggests a potential inhibitory role for BTZ on the migratory mechanisms mediated by MMPs in NMSC cells, thereby highlighting a new avenue for therapeutic intervention.

### BTZ impedes lysosome-autophagosome fusion in NMSC cells

Autophagy, a cellular process that aids in the degradation and recycling of cellular components, often steps up when apoptosis (programmed cell death) is compromised, potentially augmenting apoptosis to induce cell death. In certain scenarios, when excessively active, autophagy can instigate cell death. It has been observed that multiple autophagy elements are necessary to facilitate apoptotic factors’ initiation of cell death [53]. Moreover, several signaling pathways are instrumental in regulating both autophagy and apoptosis, indicating that these two processes share common regulatory mechanisms. For example, the expression of the DRAM gene, a direct target of the potent apoptosis inducer p53, can be enhanced by p53, thereby promoting autophagy [54]. Bearing these aspects in mind, we assessed whether BTZ significantly influences autophagy by regulating LC3B-II and p62, established autophagy markers indicative of autophagosome formation. Upon treating NMSC cell lines A431 and A388 with BTZ, we observed a transformation of LC3B-I (non-lipidated form) to LC3B-II (lipidated form) and an upregulation of p62. This suggests a hindered autophagic flux due to the accumulation of autophagosomes, a phenomenon exhibited in both cell lines post-BTZ treatment. (Supplementary Fig. 3C). Given the influential roles of p53 and Skp2 in autophagy, it is plausible that these proteins may significantly contribute to the induction of autophagy-mediated cell death. This, in turn, could act as a countermeasure against cancer cell proliferation [55].

## Discussion

Despite the availability of various chemotherapeutic drugs for the treatment of Non-Melanoma Skin Cancer (NMSC), chemoresistance remains a significant challenge [56]. BTZ (BTZ), an established anti-cancer agent, has shown efficacy against various cancer cell lines in both in vitro and in vivo models [57]. Nevertheless, due to the dearth of pertinent data, its role in managing NMSC proliferation remained unclear. Hence, our current study was designed to investigate BTZ’s mechanism of action in inhibiting the growth of NMSC cell lines.

Our data elucidates that BTZ hampers NMSC proliferation by instigating the caspase cascade, a key player in programmed cell death or apoptosis. Consistently, previous studies have reported BTZ’s efficacy in reducing cell viability across multiple cancer types, including myeloma, leukemia, melanoma, lung, and other solid tumors [58–61]. In our study, we observed an increase in annexin V/PI positive cells, a decrease in procaspase-3, and an elevation in the expression of cleaved caspase-3 and PARP post-BTZ treatment, thus underlining BTZ’s proapoptotic activity. These observations align well with previous findings [37, 62].

Balancing pro- and anti-apoptotic factors, especially in the Bcl-2 protein family, is critical for maintaining mitochondrial membrane integrity [1]. Disturbances in the balance, such as an increased Bax/Bcl-2 ratio, can provoke resistance to chemotherapy drugs. Our findings demonstrate that BTZ treatment altered the mitochondrial membrane potential, as indicated by a rise in the Bax/Bcl-2 ratio. This observation reinforces our earlier work [63, 64]. The loss of mitochondrial membrane potential may trigger caspase activation by releasing cytochrome c [65], leading to apoptosis - a pathway that was evident in our study.

Induction of apoptosis is a key strategy for tumor treatment, and p53 is a well-known proapoptotic protein. p53 has been found to transactivate a host of proapoptotic proteins, such as Bax, Bid, Puma, and p21, to promote cell death [66]. Despite these insights, there is a dearth of research on p53-dependent apoptosis in cancer therapy [67], despite evidence linking p53 expression to tumor invasiveness [68]. Importantly, human cancers like prostate cancer, colorectal cancer, melanoma, pancreatic cancer, and breast carcinoma often overexpress both Skp2 and p53 [69]. Moreover, recent evidence indicates that Skp2 regulates cellular death in various human cancers by inhibiting the p53 or p27 pathway [35, 70]. Consistent with these findings, our results show that BTZ regulates the growth and survival of NMSC cells by targeting the Skp2-p53 axis, thereby suggesting the therapeutic potential of Skp2/p53 as a target in managing NMSC.

Reactive oxygen species (ROS), a metabolic byproduct, exerts both beneficial and detrimental effects on cellular function. When ROS levels surpass a certain threshold, cellular integrity and functionality deteriorate [40]. We found that BTZ administration enhanced cellular and mitochondrial ROS in A431 and A388 cells in a dose-dependent manner, suggesting the role of ROS in BTZ-mediated apoptosis. This finding was further bolstered by the observation that ROS scavenger N-acetylcysteine (NAC) inhibited BTZ-induced caspase activation and PARP cleavage in A431 and A388 cells.

ECM remodeling, invasion, and migration, key processes in metastasis, are associated with Matrix metalloproteinase (MMP) activation. We found that BTZ downregulated MMP-1 and MMP-2 and upregulated TIMP-1, an MMP inhibitor, attenuating NMSC development and metastasis. While these findings are promising, more research is required to clarify the precise mechanisms BTZ modulates these processes.

Interestingly, p53 can act as both an inhibitor and an autophagy activator, depending on its cellular location and activity. Our results indicated that BTZ induced accumulation of LC3 and p62, two markers of autophagy, suggesting that BTZ may regulate autophagy through a mechanism involving the impairment of fusion between autophagosomes and lysosomes. This opens up new possibilities for further investigating the relationship between Skp2- p53/autophagy in NMSC cell lines.

Notably, BTZ was also found to induce apoptosis in both an in vitro cell line model and an in vivo model using *C. elegans*. We have also investigated the apoptotic effect in an in vivo model using *C. elegans*. Treatment of *C. elegans* with BTZ for 24 h leads to a significant increase in germline apoptosis. Using *C. elegans* caspase-null, *ced-3* mutants showed that the apoptotic phenotype imposed by the BTZ treatment depends on *ced-3*/ Caspase. In line with our in vitro data using cell lines, the apoptotic phenotype we observed in C. elegans treated with BTZ depended on the function of the worm’s p53, CEP-1. *C. elegans cep-1* is the functional orthologue of the human p53 activated upon inducing DNA damage response. BTZ treatment of the *cep-1* null mutants failed to induce apoptosis, indicating that the apoptotic phenotype imposed by treating BTZ induces DNA damage response in human cells as well as in *C. elegans*.

In conclusion, this study provides new insights into the mechanisms BTZ exerts its anti-cancer effects against NMSC. Despite its limitations, it highlights promising avenues for future research and has potential implications for developing more effective NMSC treatments.

## Conclusion

Our findings present BTZ as a promising anti-cancer agent for NMSC. BTZ significantly downregulated Skp2 and upregulated p53 expression in NMSC cell lines, causing impaired cellular growth and caspase-dependent apoptosis. It also disrupted mitochondrial membrane potential by increasing the Bax to Bcl-2 ratio, inducing the release of cytochrome C. BTZ additionally prompted reactive oxygen species (ROS) generation and autophagy, highlighting its multifaceted anti-cancer effects. Notably, in vivo tests on *C. elegans* showed that even low concentrations of BTZ induced germline apoptosis in worms, demonstrating its robust activity across species. Importantly, this apoptotic process was mediated through *cep-1*, a counterpart to mammalian p53, further confirming the role of the Skp2/p53 axis in BTZ’s mechanism of action. Collectively, these results underscore the potential of BTZ in NMSC treatment through its modulation of the Skp2/p53 axis, ROS production, and autophagy induction. These findings not only enhance our understanding of BTZ’s mode of action but also offer promising avenues for developing novel, alternative therapeutic strategies against NMSC. We hope these insights pave the way toward a future where targeted, less invasive treatments for skin cancer become a viable reality.

## Materials and methods

BTZ, Cell Counting Kit-8, and N-acetylcysteine (NAC) were purchased from Sigma Aldrich (St. Louis, MO, USA). z-VAD-FMK was purchased from Calbiochem (San Diego, CA, USA). Antibodies against Skp2, p21, p27, MTH1, CDK4, CDK6, caspase-9, Bcl-2, cleaved caspase-3, caspase-3, Bax, p53, Cytochrome c, PARP, Cleaved caspase-8, PH2AX were purchased from Cell Signaling Technology (Danvers, MA, USA). FITC Annexin V apoptosis detection kit I, Apo-Direct kit, Fixation/Permeabilization solution kit, BD MitoScreen (JC1), BV421 mouse anti-γH2AX (pS139), PE rabbit anti-active caspase-3, and Alexa Fluor 700 mouse anti-cleaved PARP (Asp214) antibodies were purchased from BD Biosciences (San Jose, USA). CellROXGreen was purchased from Invitrogen (Massachusetts, USA).

### Cell culture condition

The NMSC cells A431 and A388 used in this work were obtained from the American Type Culture Collection (ATCC). Each cell line was accompanied by a Certificate of Analysis, which confirmed their authentication using STR profiling and verified that they were free of mycoplasma contamination. Upon receiving the cell lines, they were thawed and grown in DMEM medium with 10% fetal bovine serum (FBS), 100 U/ml penicillin, and 100 U/ml streptomycin at 37 °C in a humidified atmosphere with 5% CO_2_ [71]. Primary human Keratinocyte were bought from PromoCell (C-12005, Heidelberg, Germany) and grew in Keratinocyte SFM media (Gibco, United States) supplemented with 0.05 mg/ml bovine pituitary extract (BPE), 0.005 µg/ml epidermal growth factor (EGF), 100 U/ml penicillin,100 μg/ml streptomycin (Gibco, United States) at 37 °C in a humidified atmosphere with 5% CO_2_ [72].

### Cell viability assay

Using the CCK-8 colorimetric method, the cell viability of the A431 and A388 NMSC cell lines that were treated with or without BTZ was tested. The number of living cells is directly linked to the amount of formazan dye that is made when WST-8 salt [2-(2-methoxy-4-nitrophenyl)-3-(4-nitrophenyl)-5-(2,4-disulfophenyl)-2H-tetrazolium] is broken down by dehydrogenase in cells. In short, 1 × 10^4^ cells were seeded into each well of a 96-well microtiter plate, and the cells were treated with increasing doses of BTZ for 48 h. At the end of 48 h, the CCK-8 solution was added according to the manufacturer’s instructions, and the plates were read at 450 nm. The percentage of live cells was calculated as mentioned earlier [63].

### Annexin V/Propidium Iodide dual staining

To evaluate the extent of apoptosis, BTZ-treated and untreated NMSC cells were subjected to Annexin V/Propidium Iodide (PI) dual staining. After 48 h of BTZ exposure, cells were gently washed with PBS and stained with fluorescein-conjugated Annexin V and PI in 1x Annexin binding buffer for 20 min in the dark. Following staining, cells were analyzed by flow cytometry, quantifying viable cells, early apoptotic, late apoptotic, and necrotic cells [73].

### Cell cycle analysis

The effect of BTZ on the cell cycle of NMSC cell lines A431 and A388 was examined after a 48-h treatment period. Cells were stained with Hoechst 33342, a fluorescent dye binding to the minor groove of DNA, to assess cell cycle phase distribution. The stained cells were subsequently analyzed using the BD LSRFortessa flow cytometer (BD Biosciences, NJ, USA) [64].

### Cell lysis and western blotting

Following 48 h of BTZ treatment, A431, and A388 cells were washed with PBS and lysed using the radioimmunoprecipitation assay (RIPA) buffer supplemented with a cocktail of protease-phosphatase inhibitors (Roche). Cell lysates were then centrifuged, and the protein-rich supernatant was collected. The total protein concentration was determined using the Rapid Gold BCA Protein assay kit (PierceTM, Thermo Scientific, Waltham, MA, USA) [73].

### siRNA transfection

For transient silencing of the Skp2 gene in A388 cells, the cells were transfected with small interfering RNA (siRNA) specifically designed to target the human Skp2 gene. The control and Skp2-targeting siRNA were synthesized and obtained from Santa Cruz Biotechnology, Inc., USA. The transfection agent of choice was Lipofectamine 2000 reagent, supplied by Invitrogen, USA. 5 × 10^5^ cells in the exponential growth phase were plated into 6-well plates. DMEM supplemented with 10% FBS and 1% penicillin-streptomycin was used as the culture medium. The cells were incubated at 37 °C in a humidified incubator containing 5% CO_2_ until they reached a confluency of approximately 60%. For the transfection process, the Lipofectamine 2000 reagent and siRNAs (80 pmols) were each diluted in Opti-MEM I Reduced Serum Medium (Invitrogen, USA) according to the manufacturer’s guidelines. Following a brief incubation period of 5 min at room temperature, the diluted Lipofectamine 2000 reagent and siRNA were mixed gently, and incubated for another 20 min to allow the formation of transfection complexes. The medium in the 6-well plates was then replaced with fresh DMEM (without antibiotics), and the Lipofectamine-siRNA complexes were added to each well. The plates were returned to the incubator for 48 h to allow for sufficient knockdown of the Skp2 gene. Following the 48-h incubation period, the cells were harvested and lysed using a suitable lysis buffer containing protease inhibitors. The protein concentration in the lysates was quantified using the BCA Protein Assay Kit (Pierce, USA). Equal amounts of protein were loaded onto an SDS-PAGE gel for separation, then transferred onto a PVDF membrane. The membrane was then incubated with a primary antibody against Skp2 and a suitable secondary antibody. The resulting bands were visualized using enhanced chemiluminescence (ECL), and the knockdown efficiency was determined.

### Measurement of active Caspase-3 and cleaved PARP

To determine the activation of apoptotic pathways, the levels of active Caspase-3 and cleaved PARP were quantified in NMSC cells after 48 h of BTZ treatment. Both treated and untreated cells were fixed and permeabilized using the BD Cytofix/Cytoperm Plus Fixation kit according to the manufacturer’s instructions. Cells were stained with 5 μL of anti-active Caspase-3-BV605 and 5 μL of anti-PARP Cleaved Form-AF700 antibodies per 1 × 10^5^ cells for 30 min in the dark. Following a wash step, cells were analyzed by flow cytometry to measure the fluorescence intensities correlating to active Caspase-3 and cleaved PARP levels [63].

### Mitochondrial membrane potential measurement

Mitochondrial membrane potential changes in A431 and A388 cells subjected to BTZ treatment were evaluated using a JC1 stain kit. This kit leverages the cationic dye JC1, which accumulates in mitochondria in a potential-dependent manner. A decrease in the red-to-green fluorescence intensity ratio indicates reduced membrane potential. Flow cytometry was used to measure the red fluorescence of each sample, reflecting the mitochondrial membrane potential (MMP) [63].

### Cytochrome c release assay

After treating A431 and A388 cells with BTZ for 48 h, cells were collected and resuspended in a hypotonic buffer. Mitochondrial and cytosolic protein fractions, as mentioned earlier, were isolated following the protocol outlined by Uddin et al. [74]. Subsequently, the cytosolic fraction of A431 and A388 cells was resolved using 12% SDS-PAGE and probed with antibodies against cytochrome c and HSP60. The presence of cytochrome c in the cytosolic fraction indicated apoptosis initiation [73].

### Reactive oxygen species and mitochondrial superoxide determination

Following BTZ treatment for 48 h, A431 and A388 cells were washed with Hank’s Balanced Salt Solution (HBSS) and stained for 20 min at 37 °C with 5 μM CellROXTM Green Reagent (Invitrogen, MA, USA) for determination of ROS at cellular level or with the MitoSOX Red Mitochondrial Superoxide Indicator (Invitrogen, MA, USA) in HBSS for determination of ROS at mitochondrial level. Cells were then subjected to flow cytometry (Ex: 488, Em: 575/26) to assess cellular/mitochondrial superoxide levels, a biomarker of oxidative stress [64].

### Fluorescence microscopy test for live/dead

Following 48 h of BTZ exposure, A431, and A388 cells were stained with calcein-AM and EthD-1, following the manufacturer’s guidelines (Thermo Fisher Scientific, Waltham, MA, USA). Calcein-AM is a viable cell marker, while EthD-1 labels dead cells. Images of the stained cells were captured using the EVOS FL Cell Imaging System (Thermo Fisher Scientific, Waltham, MA, USA) [75].

### *C. elegance* strain and maintenance

As described previously, *C. elegans* strains were maintained at 20 °C on the nematode growth media (NGM) and fed with *Escherichia* *coli* strain (OP50). The *C. elegans* N2 (Bristol) strain was used as a wild type. Other strains that were used in this study are: MD701 *bcIs39 V* [*lim-7p::ced-1::gfp + lin-15(*+*)*], KX84 *ced-3(n2452) IV; bcIs39 V* [*lim-7p::ced-1::gfp + lin-15(+)*], *and* EPD077 *bcIs39 V* [*lim-7p::ced-1::gfp + lin-15(+)*]*; cep-1(Ig12501) I*.

### *C. elegance* treatment with BTZ

To characterize the effect of BTZ in an in vivo system, synchronized L-4 stage worms were treated with 1 µM, 3 µM, and 5 µM BTZ dissolved in DMSO in the liquid culture. Worms were incubated with the corresponding BTZ concentration at 20 °C with gentle shaking. Adult worms were collected after 24 h and were scored for apoptosis induction and quantitative Real-Time PCR (qRT-PCR).

### Microscopy

#### Apoptosis scoring

A Leica 3D-Thunder Imager Fluorescent microscope equipped with Nomarski differential interference contrast (DIC) optics was used for imaging. Slides with a 3% agarose pad were prepared, to which a 5 µl of 0.5 M levamisole diluted with M9 in a 1:1 ratio was added to paralyze worms once mounted on agar pads. Various mutants carrying *ced-1::gfp* were used to score apoptosis based on CED-1::GFP expression.

#### Imaging apoptotic cells in isolated germlines

The germline was extracted and imaged as described previously with minor modifications [76]. Adult worms carrying *ced-1::gfp* were transferred to the unseeded plates to eliminate the bacteria attached to their bodies while crawling. Worms were transferred into the dissection buffer (0.2 mM Levamisole, 0.2% Tween-20 dissolved in egg buffer) on a 22 × 22 mm coverslip and were dissected near their pharynx or tail to extract the germline using a 23 Gauge syringe needle. The germlines were then fixed using fixation buffer (4% formaldehyde, 0.2 mM Tween-20 dissolved in egg buffer) and gently placed on a Poly-l-lysine coated slide. Slides were imaged using a 40x objective by capturing 0.3 µm z stacks. Z stacks were merged using maximum projection to export images.

### Randomization and blinding

No randomization or blinding was used in the current study.

### Statistical analysis

All statistical analyses and graphical presentations were performed using GraphPad Prism software (version 9.5.1). For inter-group comparisons, the mean values were statistically analyzed using a two-way ANOVA test. This test was used to determine if there were any significant differences between the mean values of the various groups being compared. The calculated p-value was used to establish the level of significance. In this study, a p-value less than 0.05 (*p < 0.05) was considered statistically significant. Greater significance levels were denoted as **p < 0.01, ***p < 0.001 and ****p < 0.0001. The graphs present the mean values derived from three independent experiments, accompanied by error bars depicting each group’s variability. These error bars represent either the standard deviation (SD) or the standard error of the mean (SEM), as deemed suitable for the experiment. By incorporating SD or SEM, the graph visually represents the data’s consistency and reliability, enabling a clearer understanding of the experimental outcomes and the extent of variation within each group.

## Supplementary information

Supplementary Figure 1

Supplementary Figure 2A

Supplementary Figure 2B

Supplementary Figure 3

Legends for supplementary figures

Supplementary File 1

Supplementary File 2

## Data Availability

The datasets used and/or analyzed during the current study are available from the corresponding author upon reasonable request.
