# Supplementary figures and images for: Bortezomib exerts its anti-cancer activity through the regulation of Skp2/p53 axis in non-melanoma skin cancer cells and C. elegans
Source: Cell Death Discov. 2024 May 9;10:225. doi: 10.1038/s41420-024-01992-7 (PMC11082213; doi:10.1038/s41420-024-01992-7)

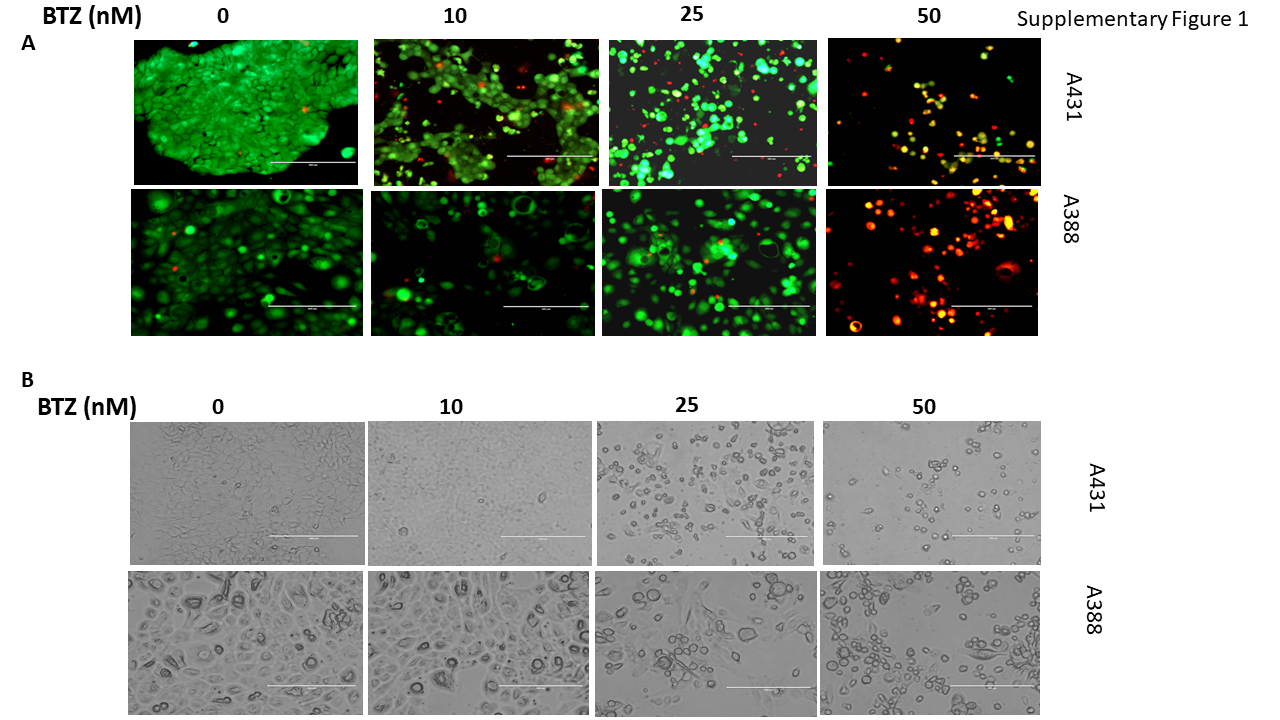

Supplement: Supplementary file 1 — Supplementary Figure 1 [file 41420_2024_1992_MOESM1_ESM.tif]

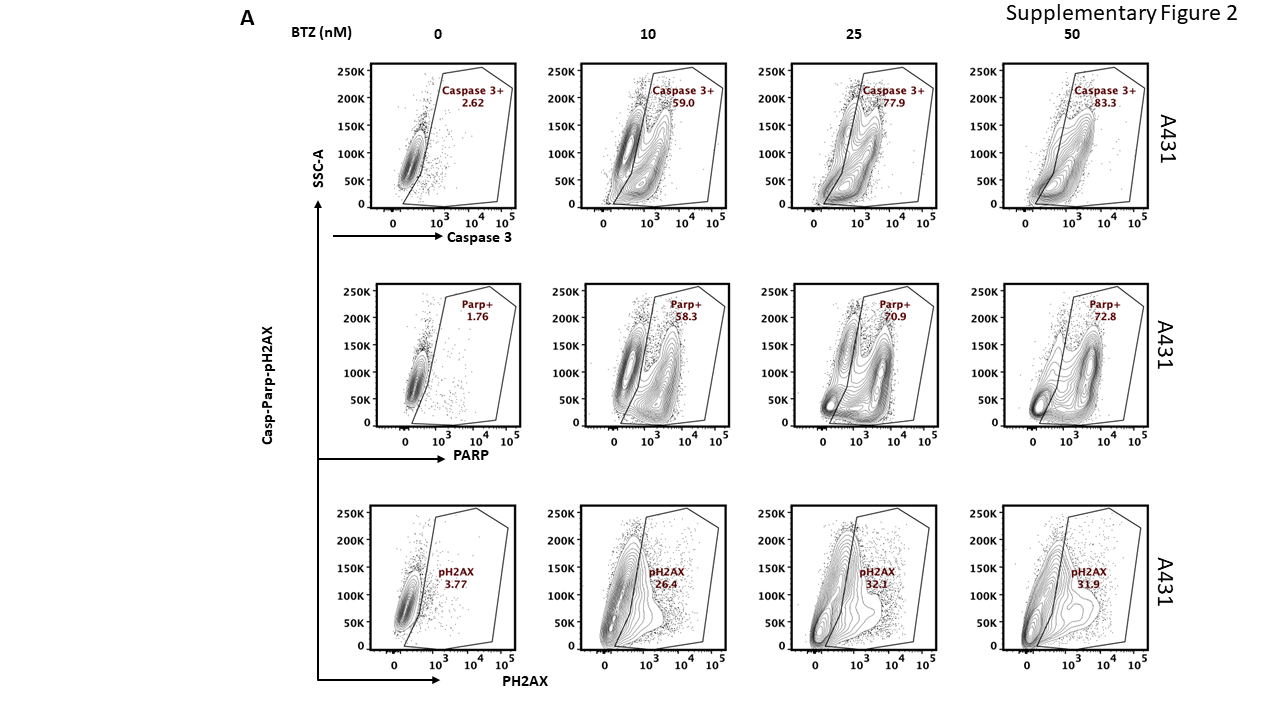

Supplement: Supplementary file 2 — Supplementary Figure 2A [file 41420_2024_1992_MOESM2_ESM.tif]

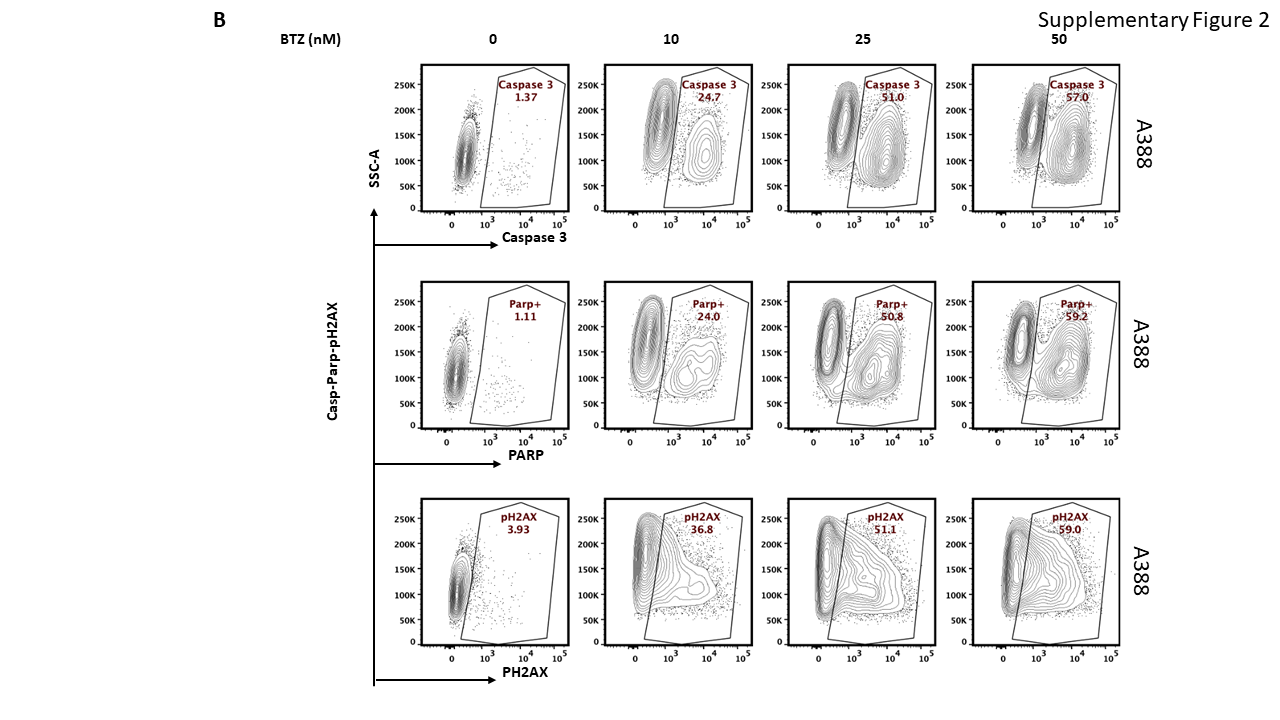

Supplement: Supplementary file 3 — Supplementary Figure 2B [file 41420_2024_1992_MOESM3_ESM.tif]

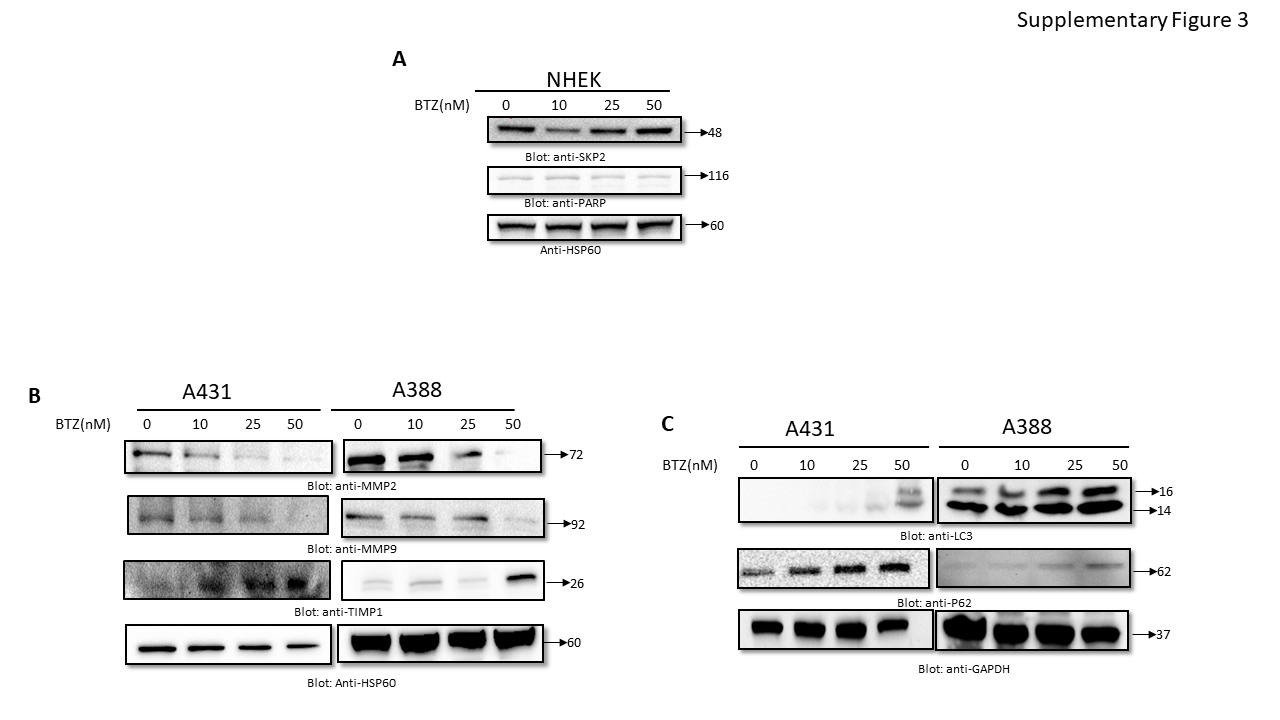

Supplement: Supplementary file 4 — Supplementary Figure 3 [file 41420_2024_1992_MOESM4_ESM.tif]
