# Supplementary material for: Bortezomib exerts its anti-cancer activity through the regulation of Skp2/p53 axis in non-melanoma skin cancer cells and C. elegans
Source: Cell Death Discov. 2024 May 9;10:225. doi: 10.1038/s41420-024-01992-7 (PMC11082213; doi:10.1038/s41420-024-01992-7)
Supplement: Supplementary file 5 — Legends for supplementary figures [file 41420_2024_1992_MOESM5_ESM.docx]

**Legends for Supplementary Figures**

**Supplementary Figure 1**: Anti-proliferative effects of BTZ on NMSC cells. (A) A431 and A388 cells were treated with BTZ in dose dependent manner for 48 h. Post treatment live/dead assay was carried out as per manufacturer protocol. Green indicates live cells and red indicates non-viable cells. (B) Morphology changes due to apoptosis in NMSC cells induced by BTZ.

**Supplementary Figure 2**: BTZ induced caspase activation in NMSC cells. (A, B) A431 and A388 cells were treated with BTZ in dose dependent manner for 48 h. BTZ mediated caspase cascade activation followed by DNA double strand breakage in A431 and A388 cells was analyzed by flow cytometry.

**Supplementary Figure 3**: Effect of BTZ on human normal keratinocytes (NHEK). (A) BTZ treatment did not cause any changes in expression levels of Skp2 and PARP expression. NHEK cells were treated with increasing doses of BTZ for 48 h, as indicated. After cell lysis, equal amounts of proteins were separated by SDS–PAGE, transferred to PVDF membrane, and immunoblotted with antibodies against Skp2 and PARP and HSP60. (B) BTZ mediated effects on matrix metalloproteinase. A431 and A388 cells were treated with increasing doses of BTZ for 48 h, as indicated. After cell lysis, equal amounts of proteins were separated by SDS–PAGE, transferred to PVDF membrane, and immunoblotted with antibodies against MMP-2, MMP-9, TIMP1, GAPDH and HSP60. Bortezomib upregulates autophagosome formation in NMSC cells. (C) BTZ Impedes Lysosome-Autophagosome Fusion in NMSC Cells. A431 and A388 were treated with or without BTZ in dose-dependent manner for 48 h, followed by immunoblotting to determine LC3B-II and p62 levels. The original western blots and quantification graphs can be found as Supplementary Files 1 and 2 respectively.
