# Supplementary material for: Bortezomib exerts its anti-cancer activity through the regulation of Skp2/p53 axis in non-melanoma skin cancer cells and C. elegans
Source: Cell Death Discov. 2024 May 9;10:225. doi: 10.1038/s41420-024-01992-7 (PMC11082213; doi:10.1038/s41420-024-01992-7)

Figure 1D

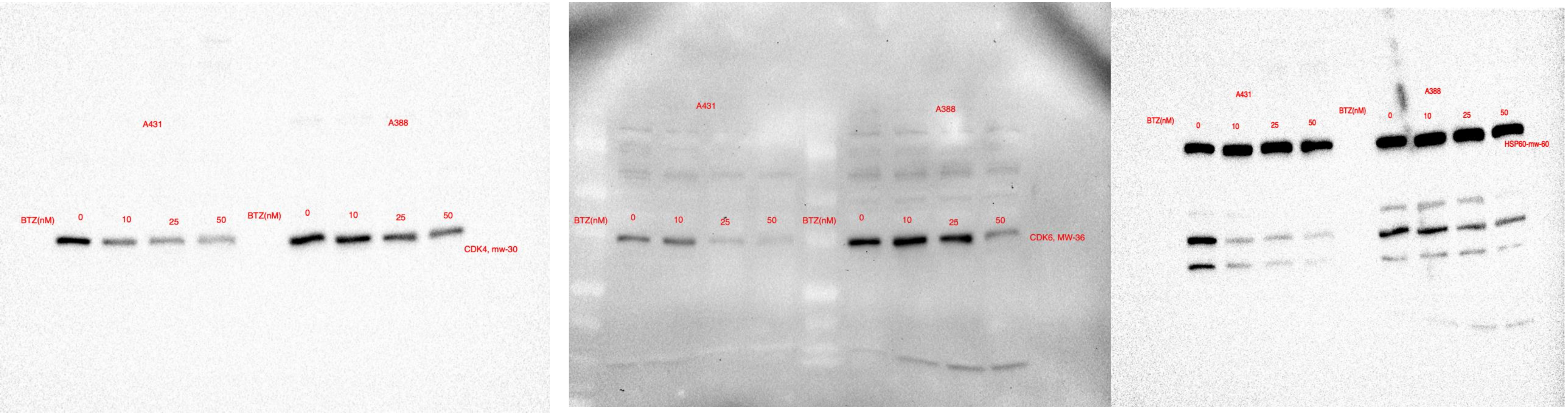
A431
A388
BTZ(nM)
0
10
25
50
BTZ(nM)
0
10
25
50
CDK4, mw-30
A431
A388
BTZ(nM)
0
10
25
50
BTZ(nM)
0
10
25
50
CDK6, MW-36
A431
A388
BTZ(nM)
0
10
25
50
BTZ(nM)
0
10
25
50
HSP60-mw-60

Figure 2C

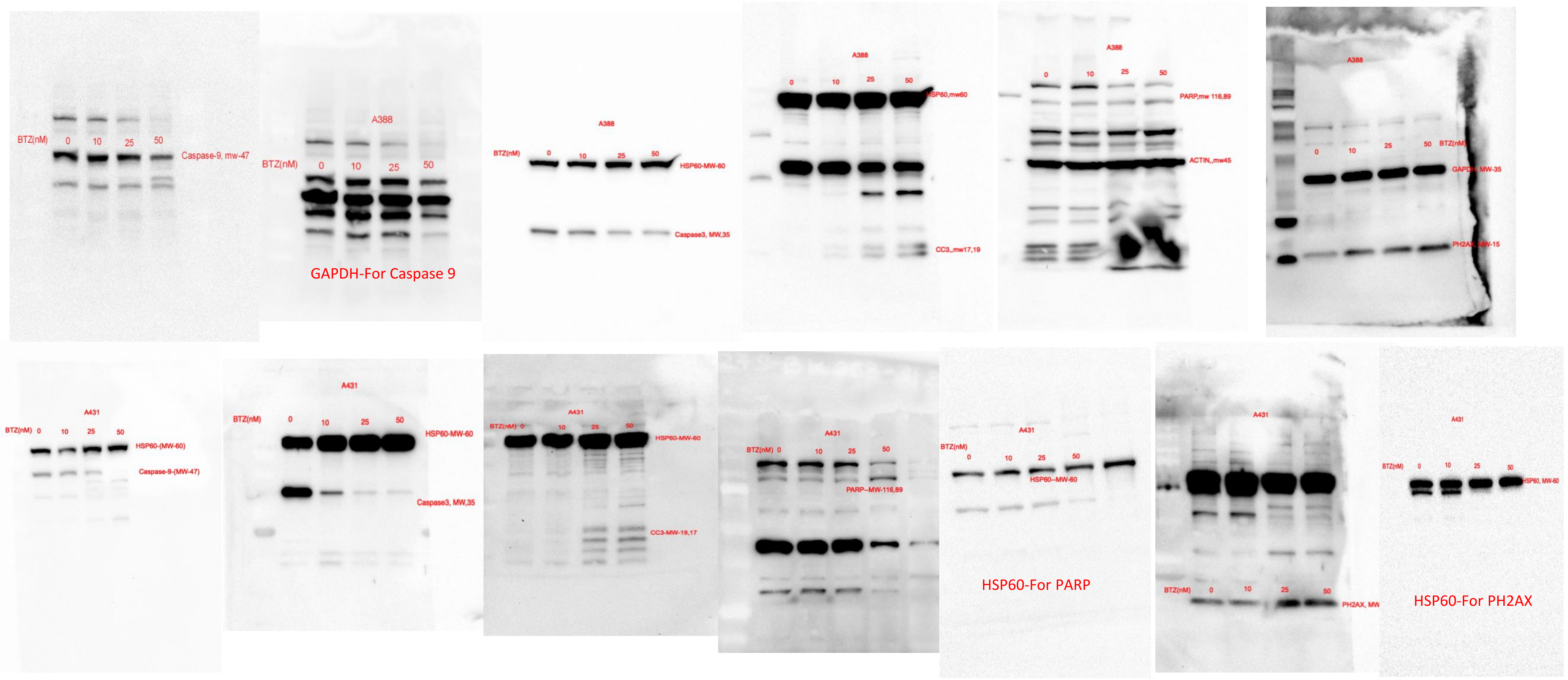

BTZ(nM) 0 10 25 50
Caspase-9, mw-47
A388
BTZ(nM) 0 10 25 50
GAPDH-For Caspase 9
A388
BTZ(nM) 0 10 25 50
HSP60-MW-60
Caspase3, MW,35
A388
0 10 25 50
HSP60,mw60
CC3,,mw17,19
A388
0 10 25 50
PARP,mw 116,89
ACTIN,,mw45
A388
0 10 25 50 BTZ(nM)
GAPDH, MW-35
PH2AX, MW-15
A431
BTZ(nM) 0 10 25 50
HSP60-(MW-60)
Caspase-9-(MW-47)
A431
BTZ(nM) 0 10 25 50
HSP60-MW-60
Caspase3, MW,35
A431
BTZ(nM) 0 10 25 50
HSP60-MW-60
CC3-MW-19,17
A431
BTZ(nM) 0 10 25 50
PARP--MW-116,89
A431
BTZ(nM) 0 10 25 50
HSP60--MW-60
HSP60-For PARP
A431
BTZ(nM) 0 10 25 50
PH2AX, MW
A431
BTZ(nM) 0 10 25 50
HSP60, MW-60
HSP60-For PH2AX

Figure 3C

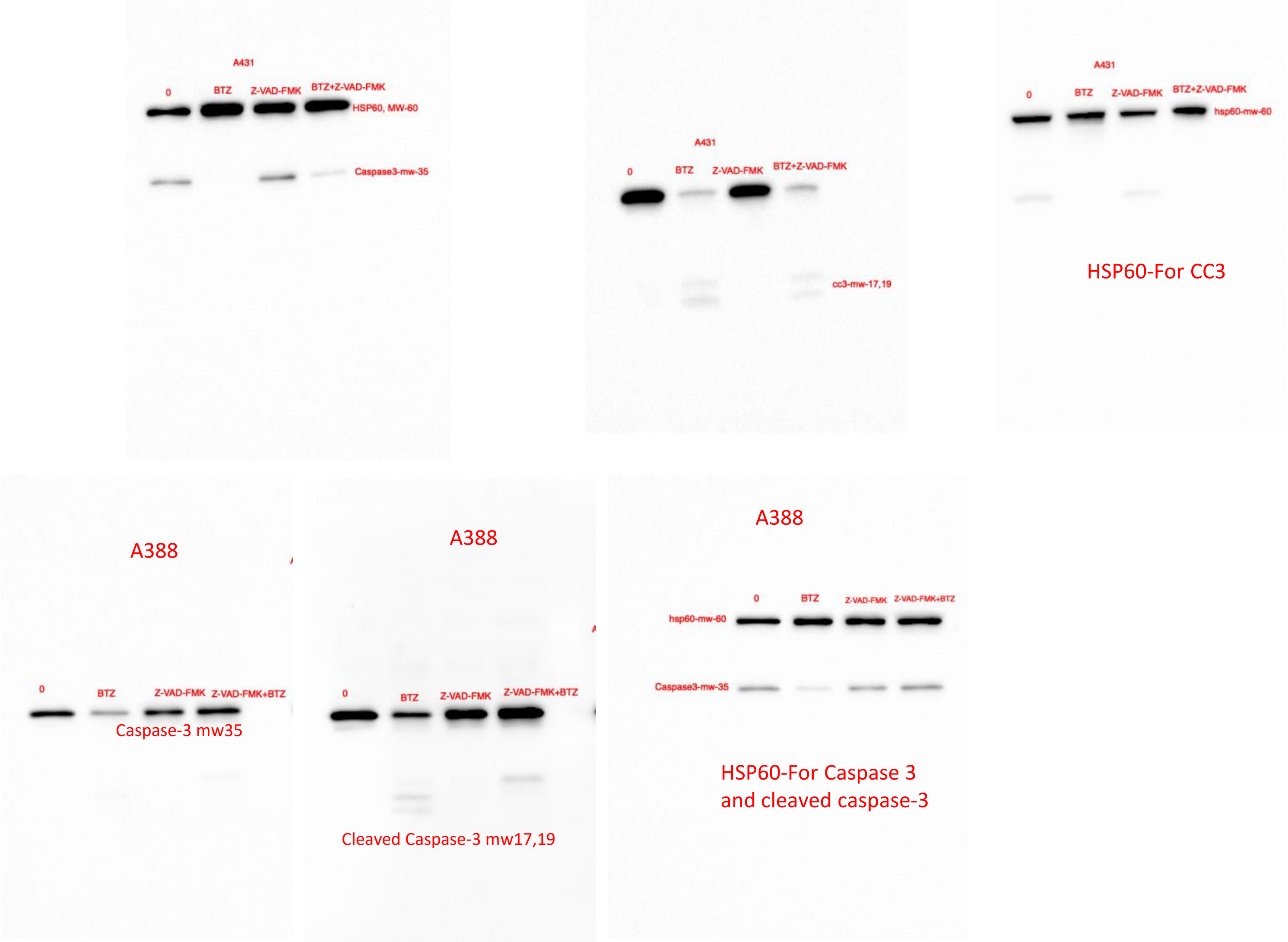
A431
0
BTZ
Z-VAD-FMK
BTZ+Z-VAD-FMK
HSP60, MW-60
Caspase3-mw-35
A431
0
BTZ
Z-VAD-FMK
BTZ+Z-VAD-FMK
cc3-mw-17,19
A431
0
BTZ
Z-VAD-FMK
BTZ+Z-VAD-FMK
hsp60-mw-60
HSP60-For CC3
A388
0
BTZ
Z-VAD-FMK
Z-VAD-FMK+BTZ
Caspase-3 mw35
A388
0
BTZ
Z-VAD-FMK
Z-VAD-FMK+BTZ
Cleaved Caspase-3 mw17,19
A388
0
BTZ
Z-VAD-FMK
Z-VAD-FMK+BTZ
hsp60-mw-60
Caspase3-mw-35
HSP60-For Caspase 3
and cleaved caspase-3

Figure 4A

A431

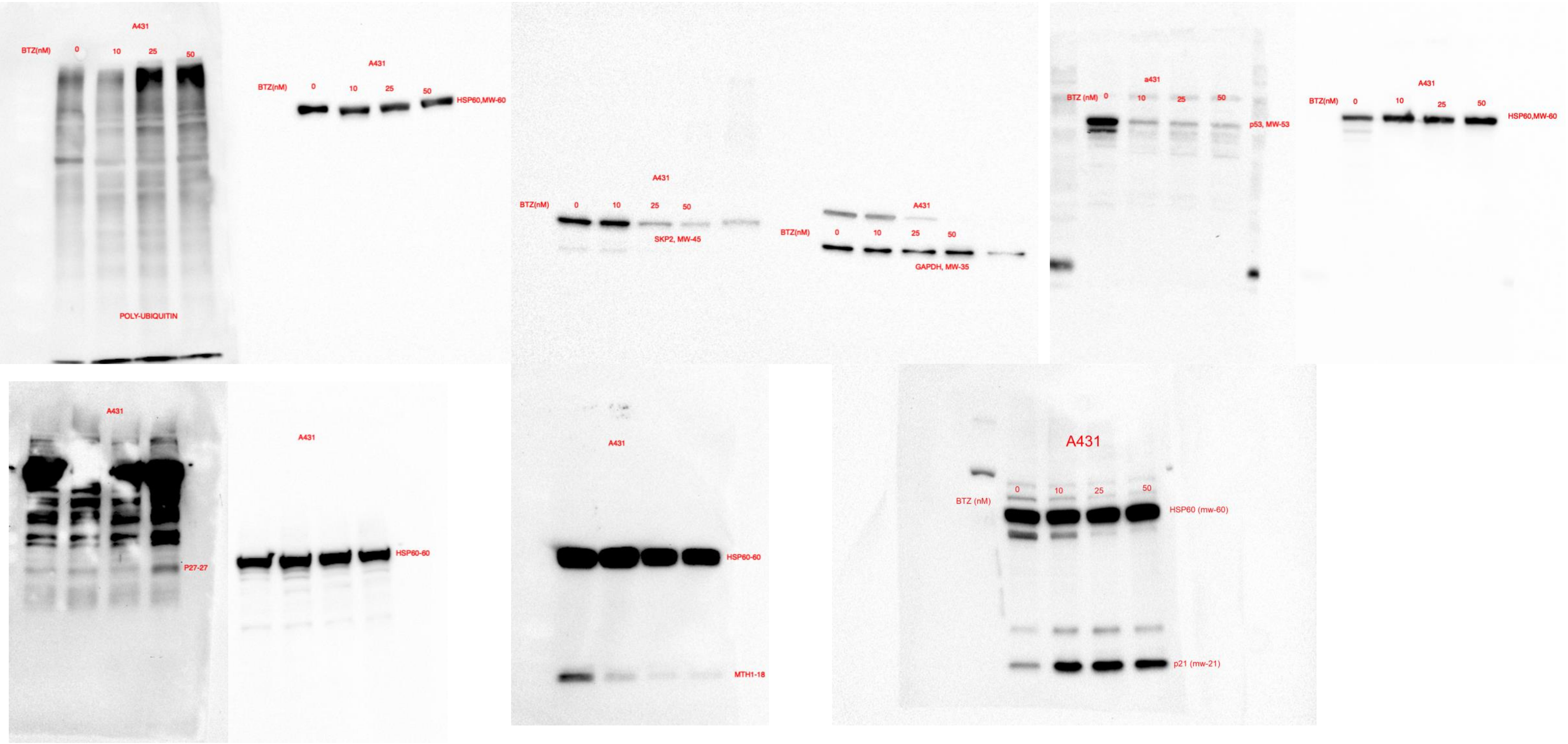

Figure 4A-A388

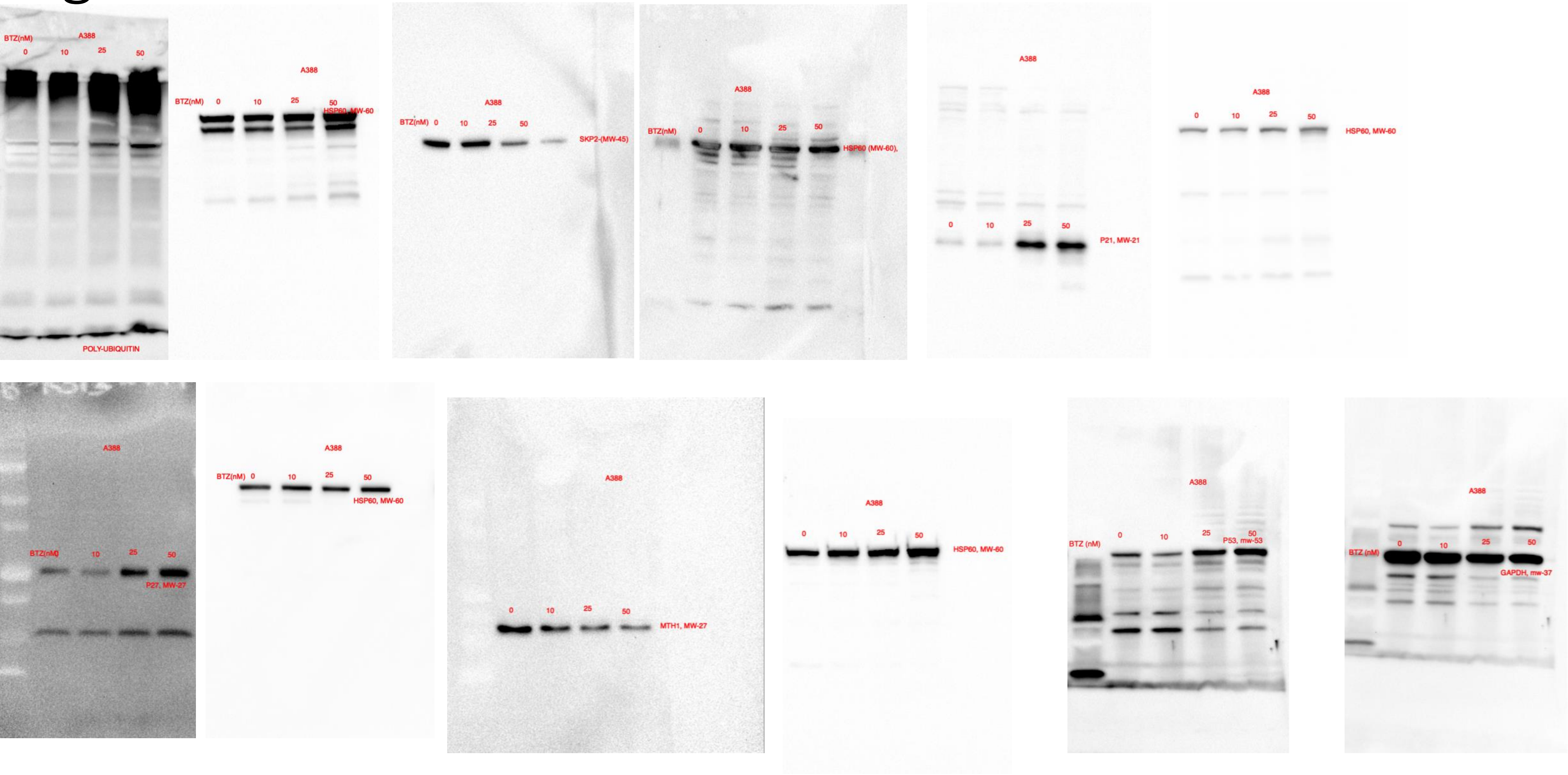

Figure 4B

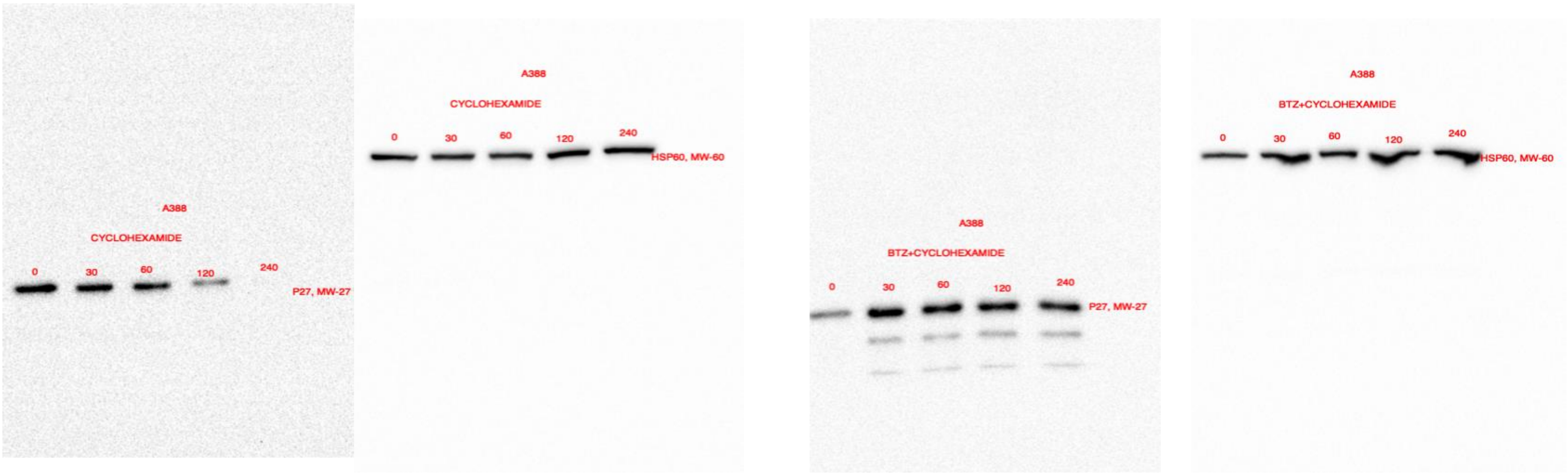

Figure 4C

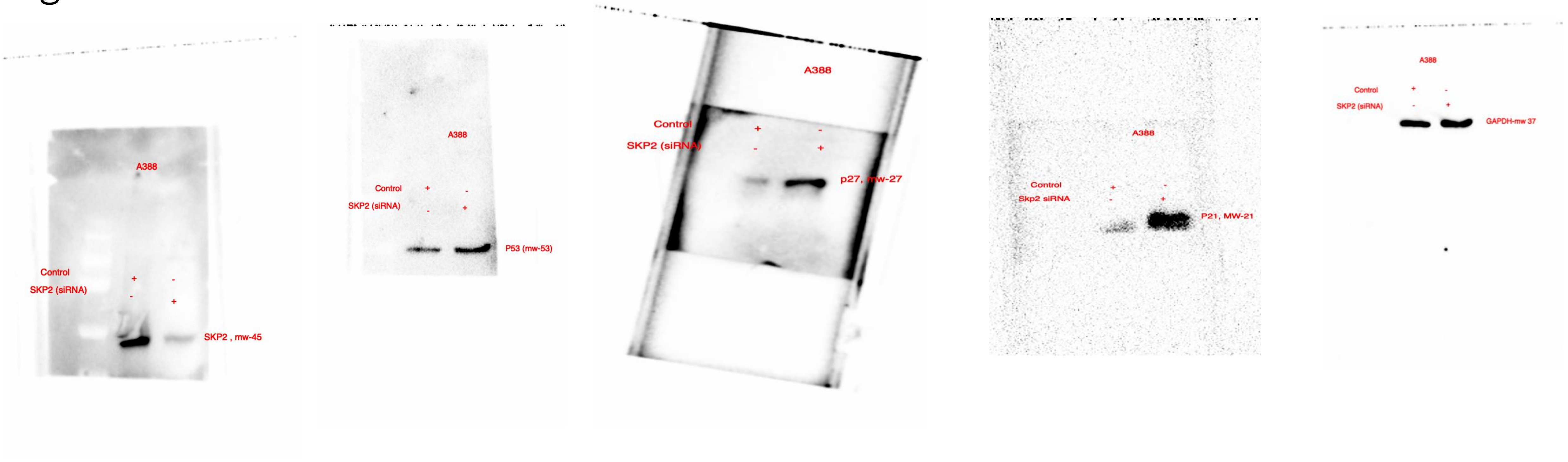

Figure 5A-A431

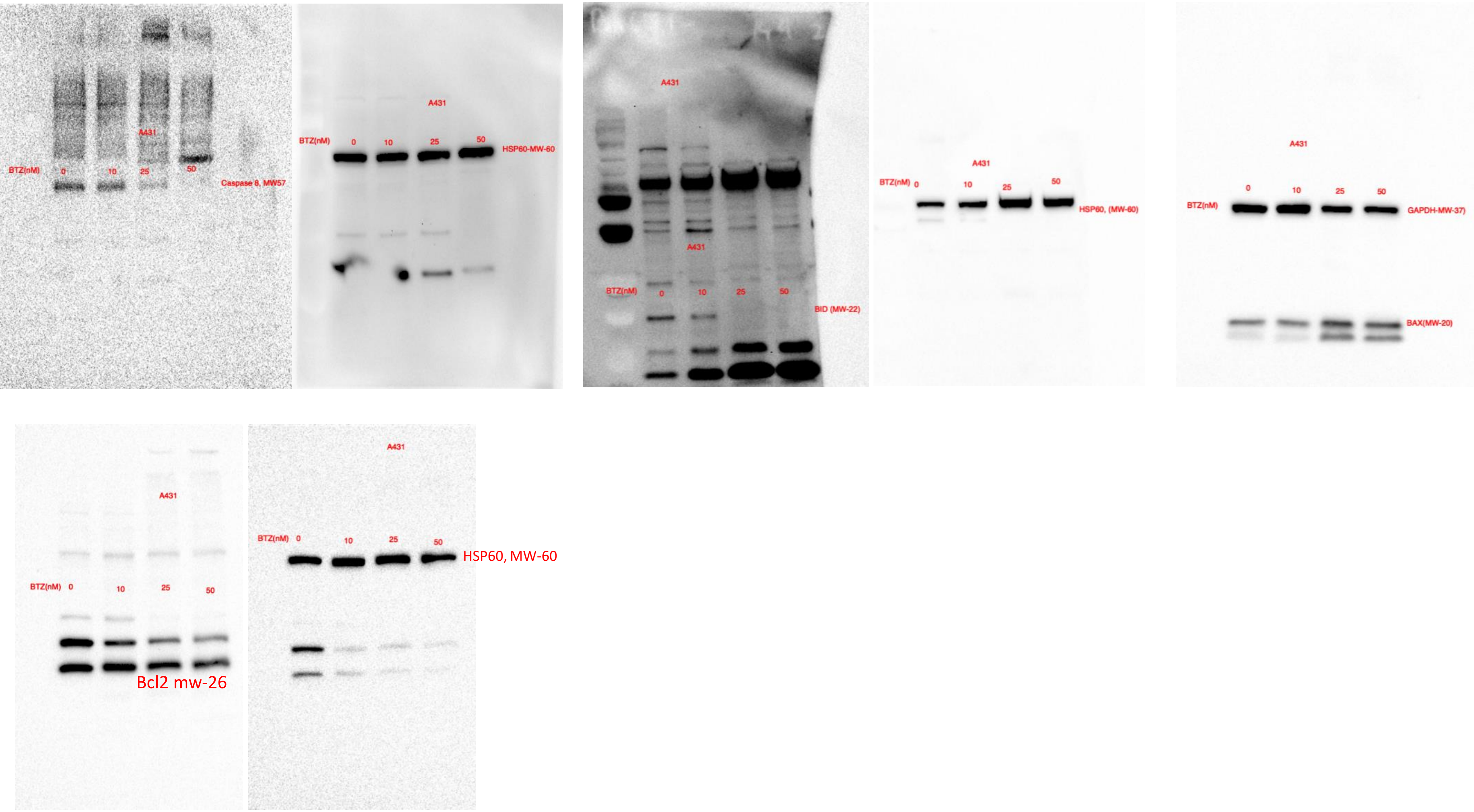

Figure 5A-A388

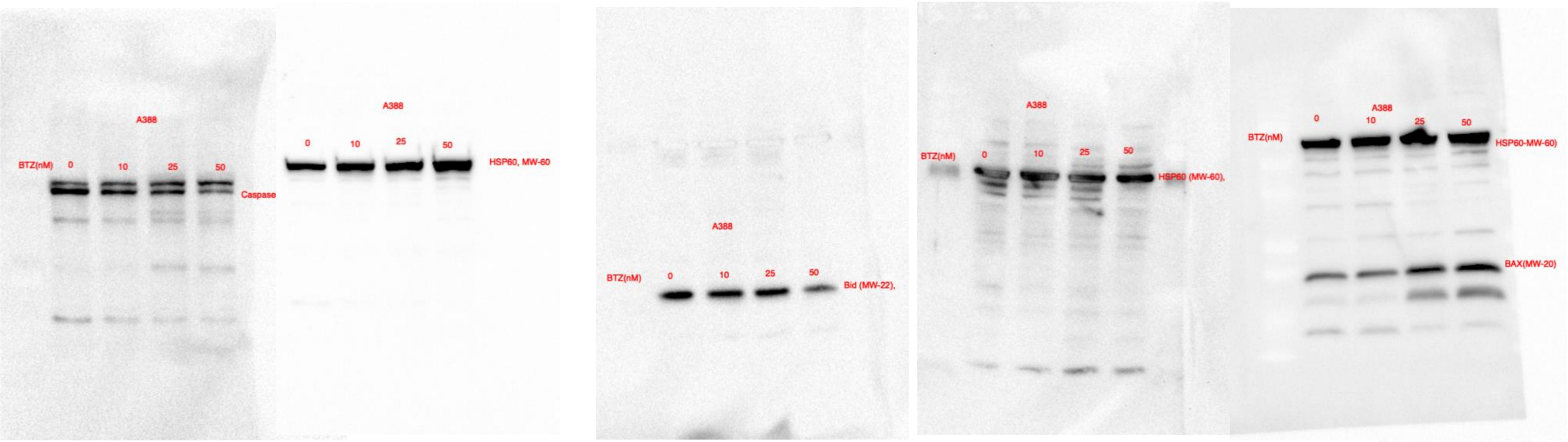

Figure 5D-A431 and A388

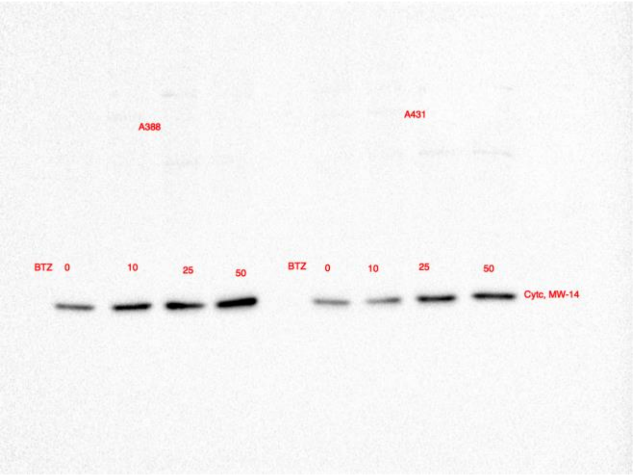

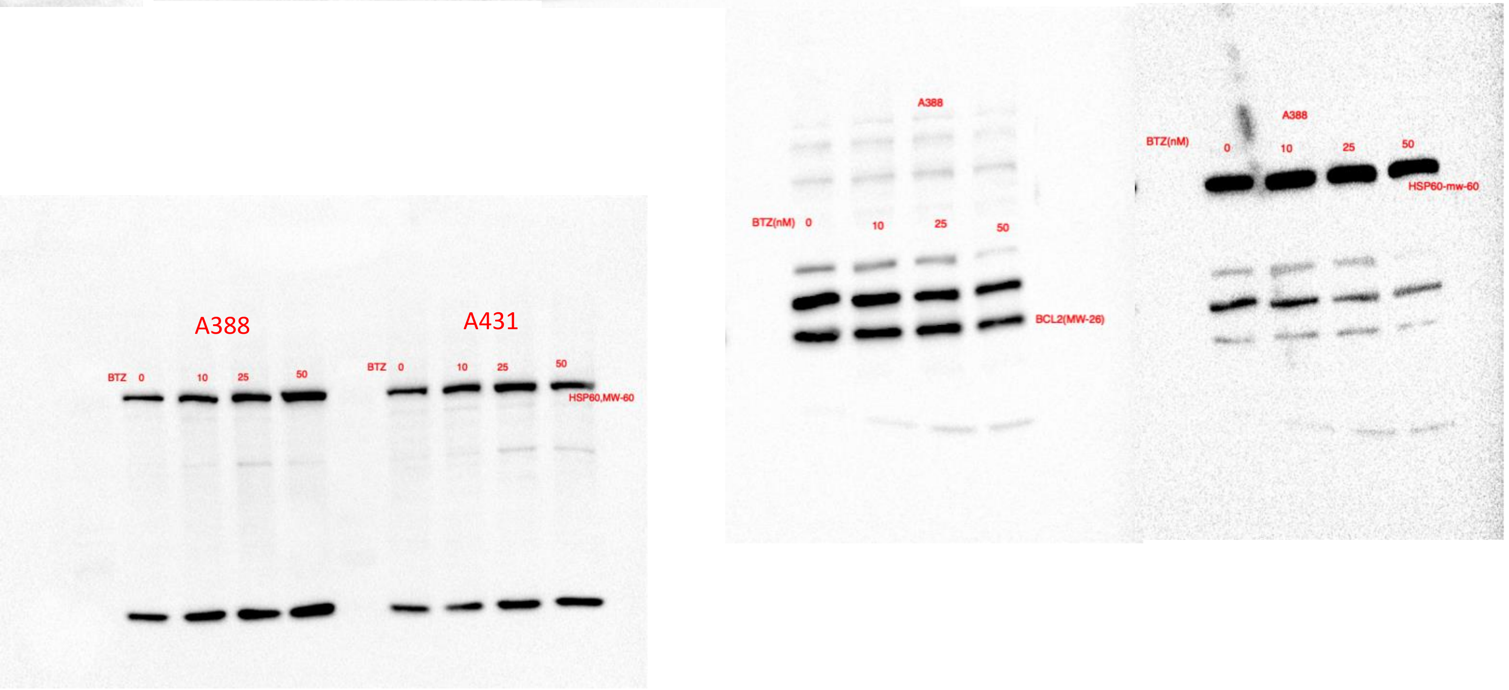

Figure 7D

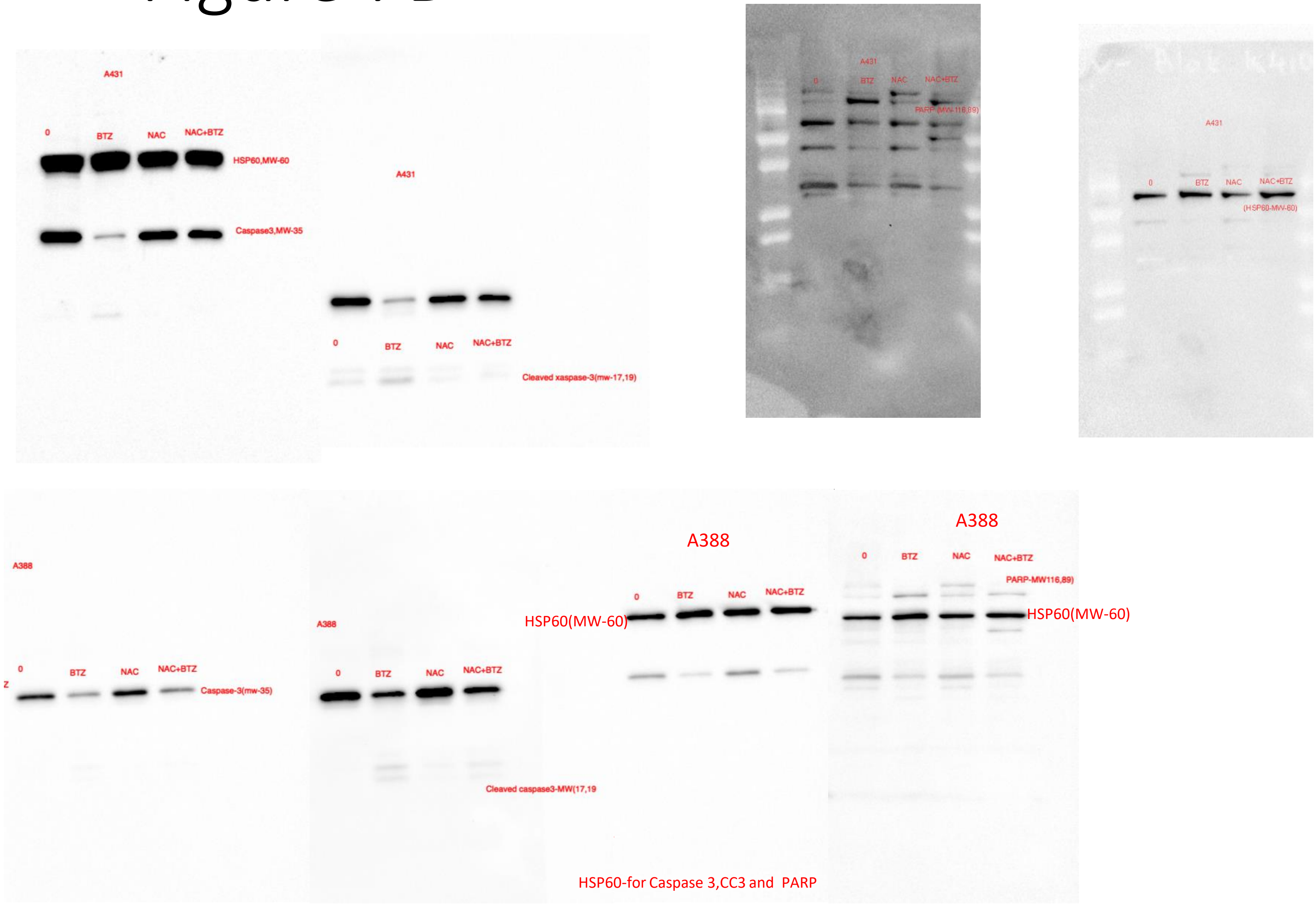
A431
0
BTZ
NAC
NAC+BTZ
HSP60,MW-60
Caspase3,MW-35
A431
0
BTZ
NAC
NAC+BTZ
Cleaved xaspase-3(mw-17,19)
A431
0
BTZ
NAC
NAC+BTZ
PARP (MW-116,89)
A431
0
BTZ
NAC
NAC+BTZ
(HSP60-MW-60)
A388
0
BTZ
NAC
NAC+BTZ
Caspase-3(mw-35)
A388
0
BTZ
NAC
NAC+BTZ
Cleaved caspase3-MW(17,19
A388
HSP60(MW-60)
0
BTZ
NAC
NAC+BTZ
A388
0
BTZ
NAC
NAC+BTZ
PARP-MW116,89)
HSP60(MW-60)
HSP60-for Caspase 3,CC3 and PARP

Supplementary Figure 3

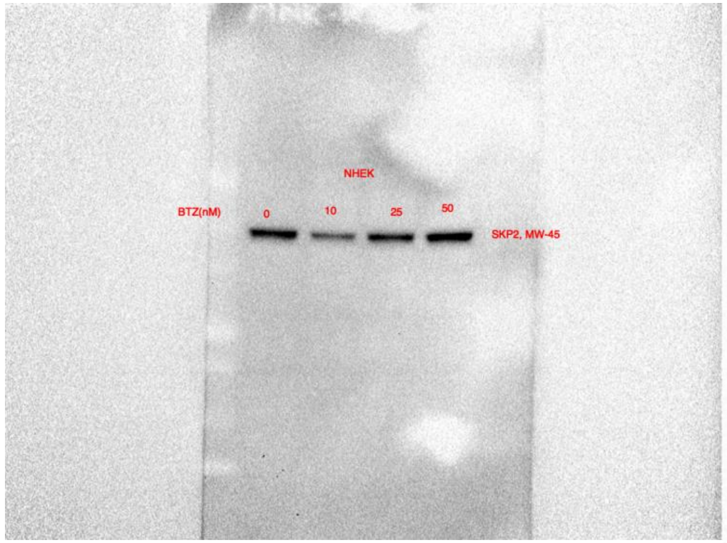

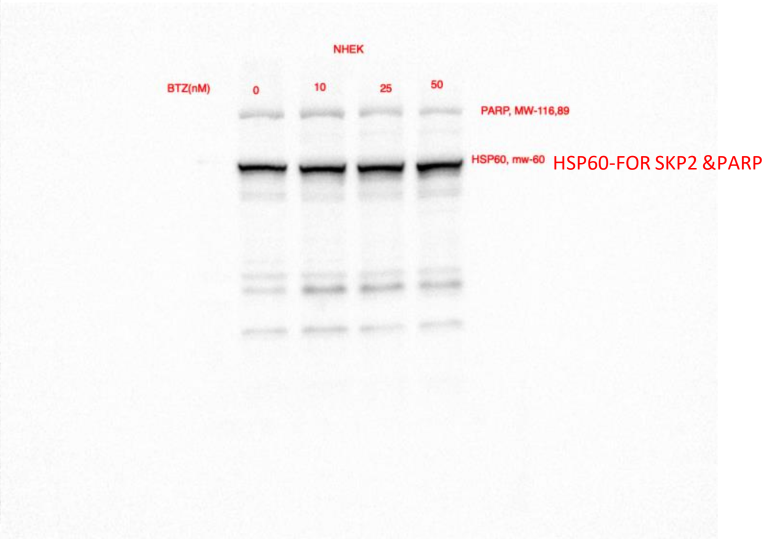

Supplementary Figure 3B

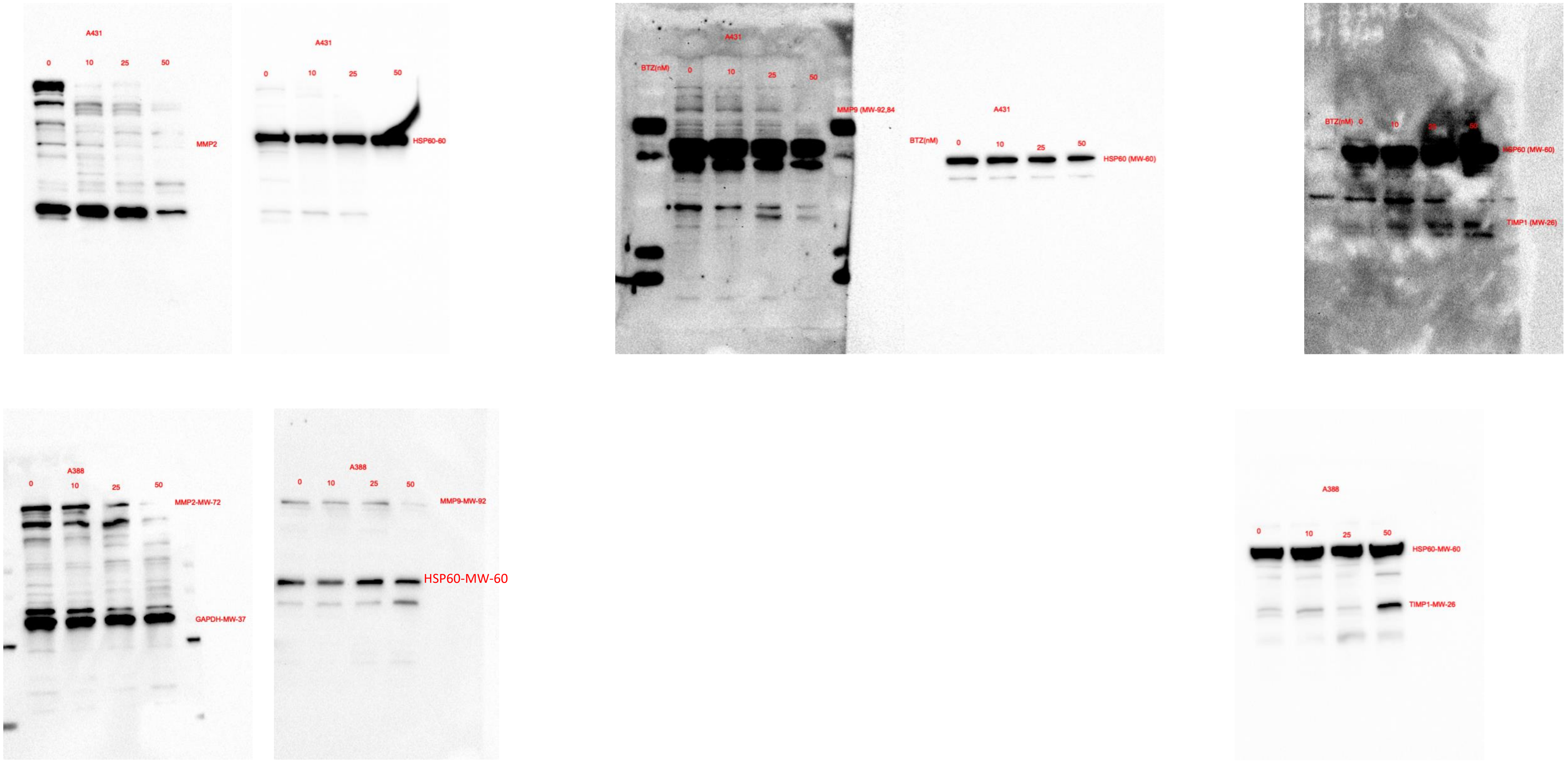

Supplementary Figure 3C

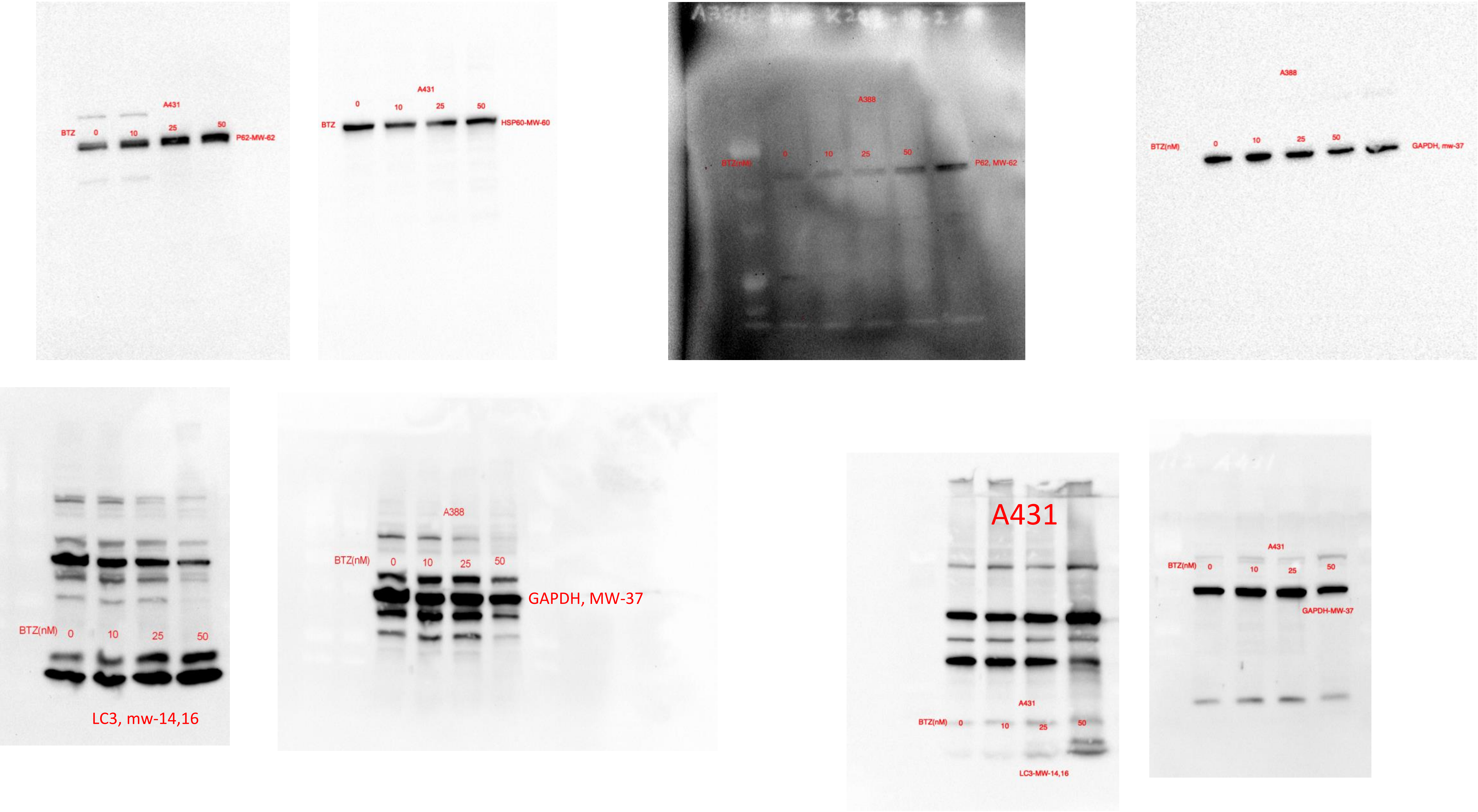

Supplement: Supplementary file 6 — Supplementary File 1 [file 41420_2024_1992_MOESM6_ESM.pdf]
