# Supplementary material for: Bortezomib exerts its anti-cancer activity through the regulation of Skp2/p53 axis in non-melanoma skin cancer cells and C. elegans
Source: Cell Death Discov. 2024 May 9;10:225. doi: 10.1038/s41420-024-01992-7 (PMC11082213; doi:10.1038/s41420-024-01992-7)

Figure 1D

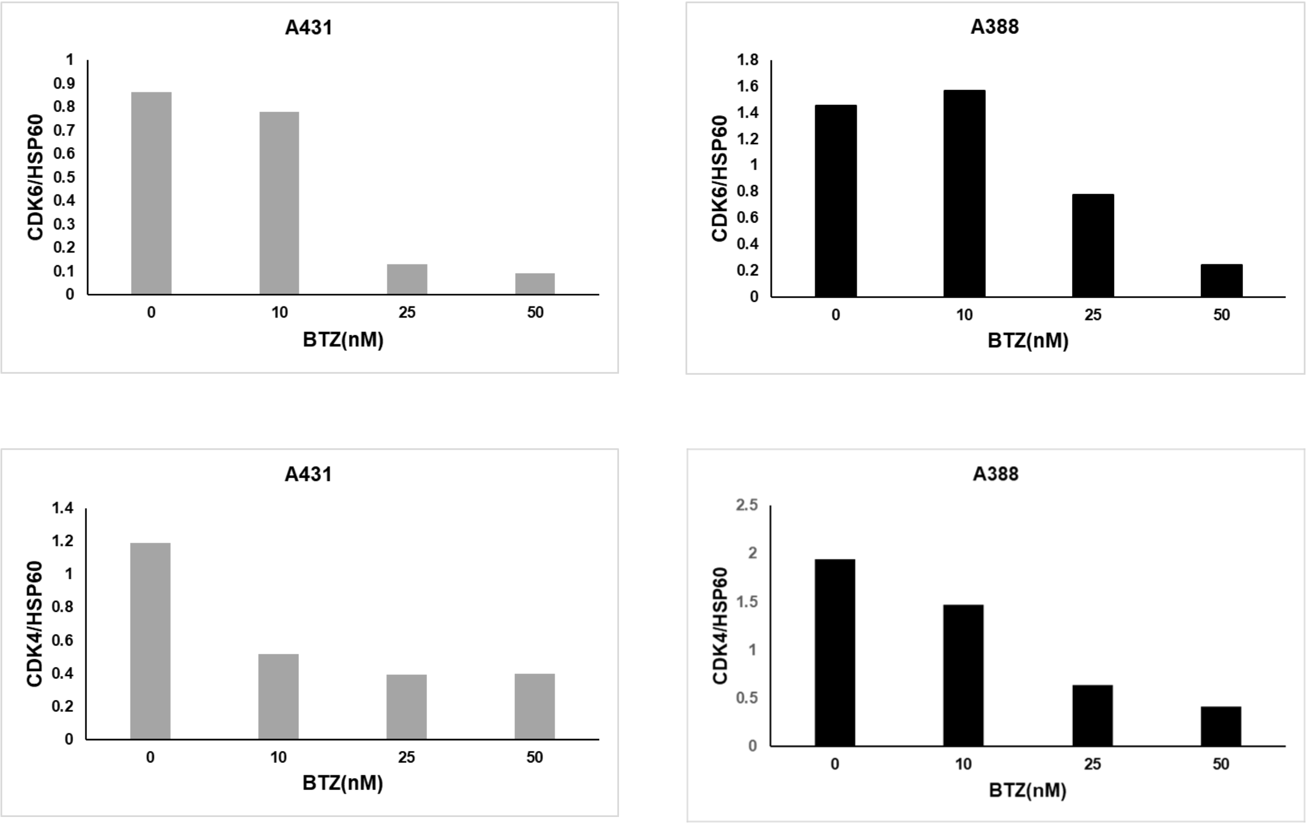
A431
CDK6/HSP60
1
0.9
0.8
0.7
0.6
0.5
0.4
0.3
0.2
0.1
0
0
10
25
50
BTZ(nM)
A388
CDK6/HSP60
1.8
1.6
1.4
1.2
1
0.8
0.6
0.4
0.2
0
0
10
25
50
BTZ(nM)
A431
CDK4/HSP60
1.4
1.2
1
0.8
0.6
0.4
0.2
0
0
10
25
50
BTZ(nM)
A388
CDK4/HSP60
2.5
2
1.5
1
0.5
0
0
10
25
50
BTZ(nM)

Figure 2C

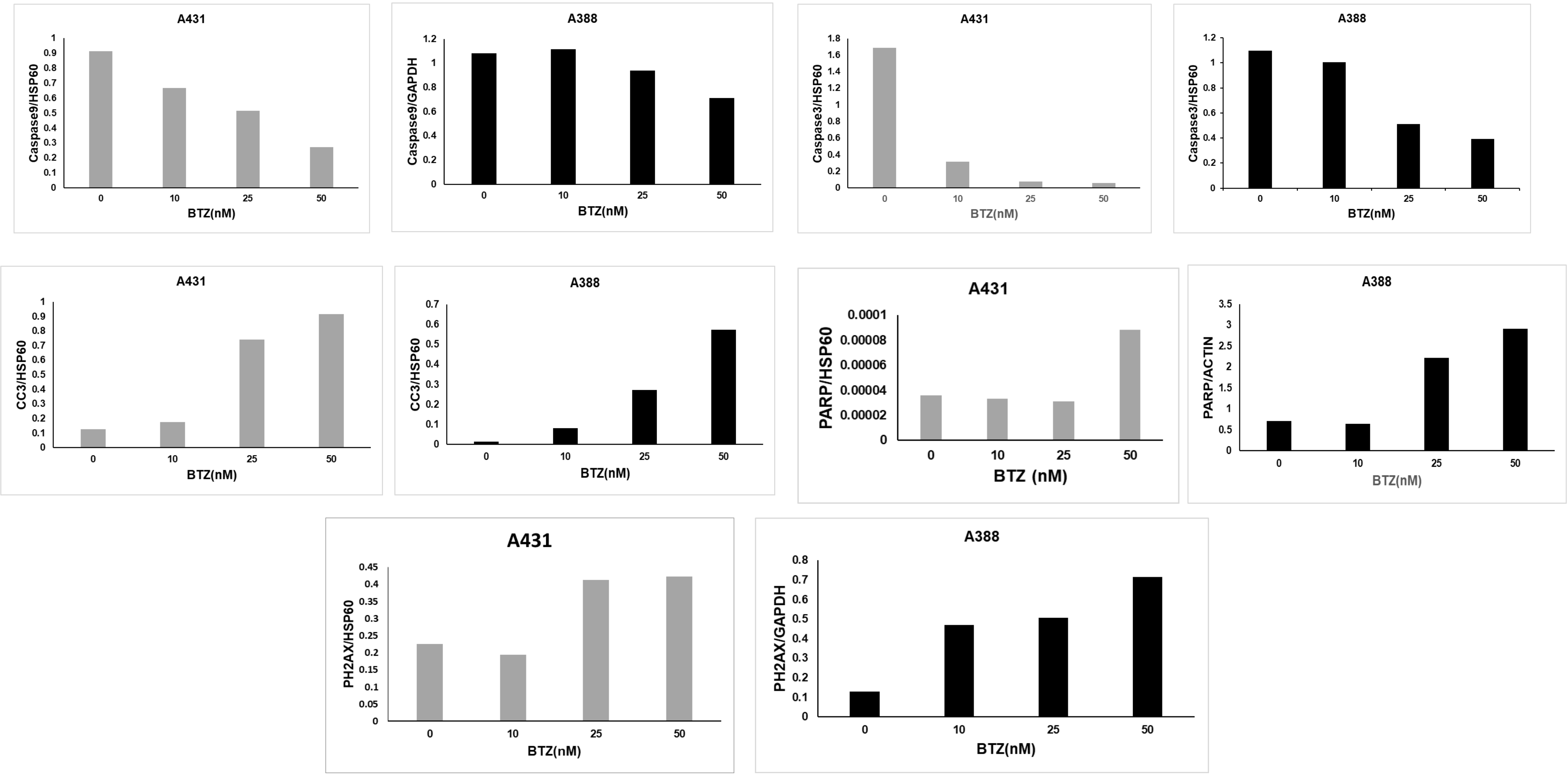
A431
Caspase9/HSP60
BTZ(nM)
A388
Caspase9/GAPDH
BTZ(nM)
A431
Caspase3/HSP60
BTZ(nM)
A388
Caspase3/HSP60
BTZ(nM)
A431
CC3/HSP60
BTZ(nM)
A388
CC3/HSP60
BTZ(nM)
A431
PARP/HSP60
BTZ (nM)
A388
PARP/ACTIN
BTZ(nM)
A431
PH2AX/HSP60
BTZ(nM)
A388
PH2AX/GAPDH
BTZ(nM)

Figure 3C

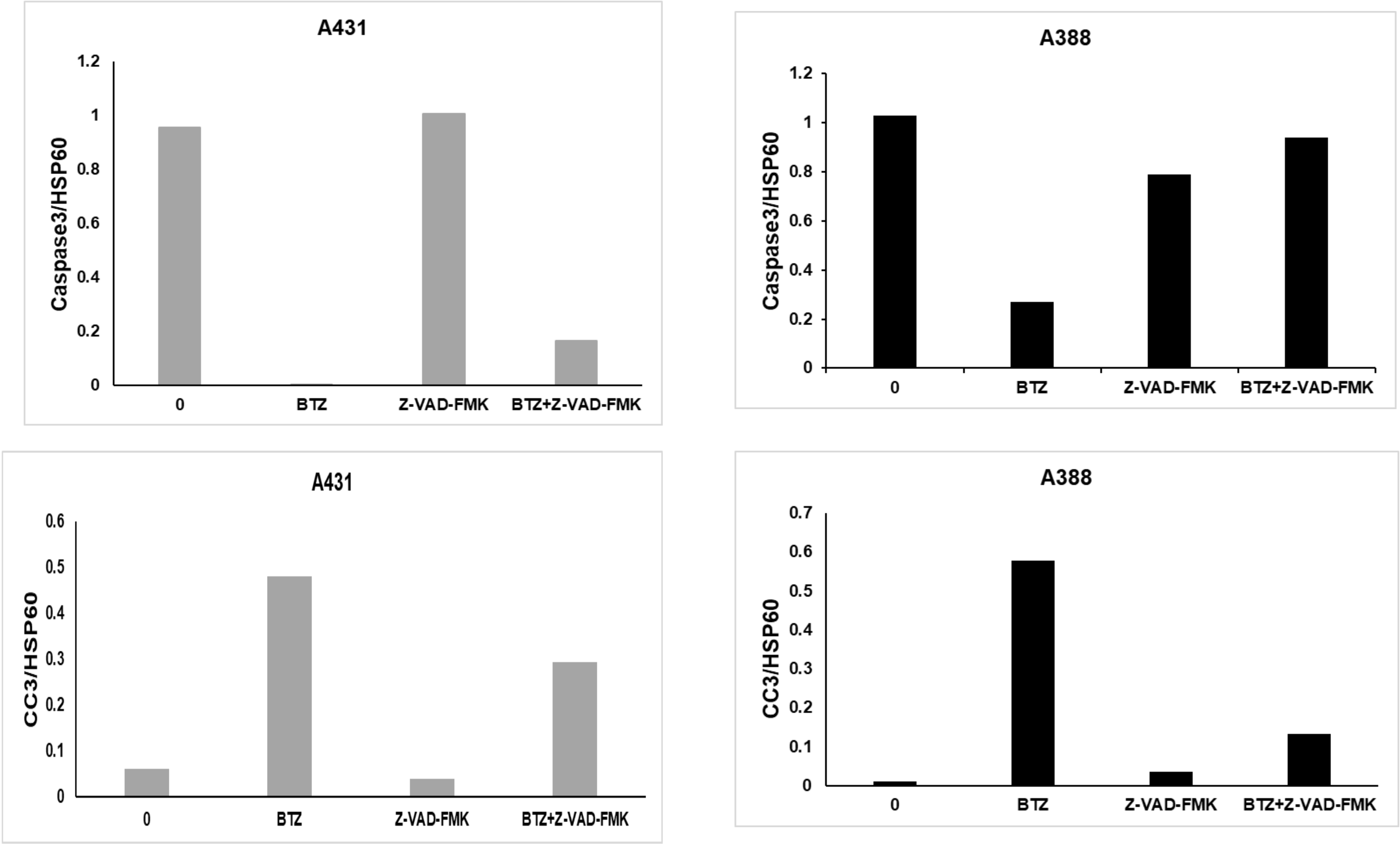
A431
Caspase3/HSP60
1.2
1
0.8
0.6
0.4
0.2
0
0
BTZ
Z-VAD-FMK
BTZ+Z-VAD-FMK
A388
Caspase3/HSP60
1.2
1
0.8
0.6
0.4
0.2
0
0
BTZ
Z-VAD-FMK
BTZ+Z-VAD-FMK
A431
CC3/HSP60
0.6
0.5
0.4
0.3
0.2
0.1
0
0
BTZ
Z-VAD-FMK
BTZ+Z-VAD-FMK
A388
CC3/HSP60
0.7
0.6
0.5
0.4
0.3
0.2
0.1
0
0
BTZ
Z-VAD-FMK
BTZ+Z-VAD-FMK

Figure 4A

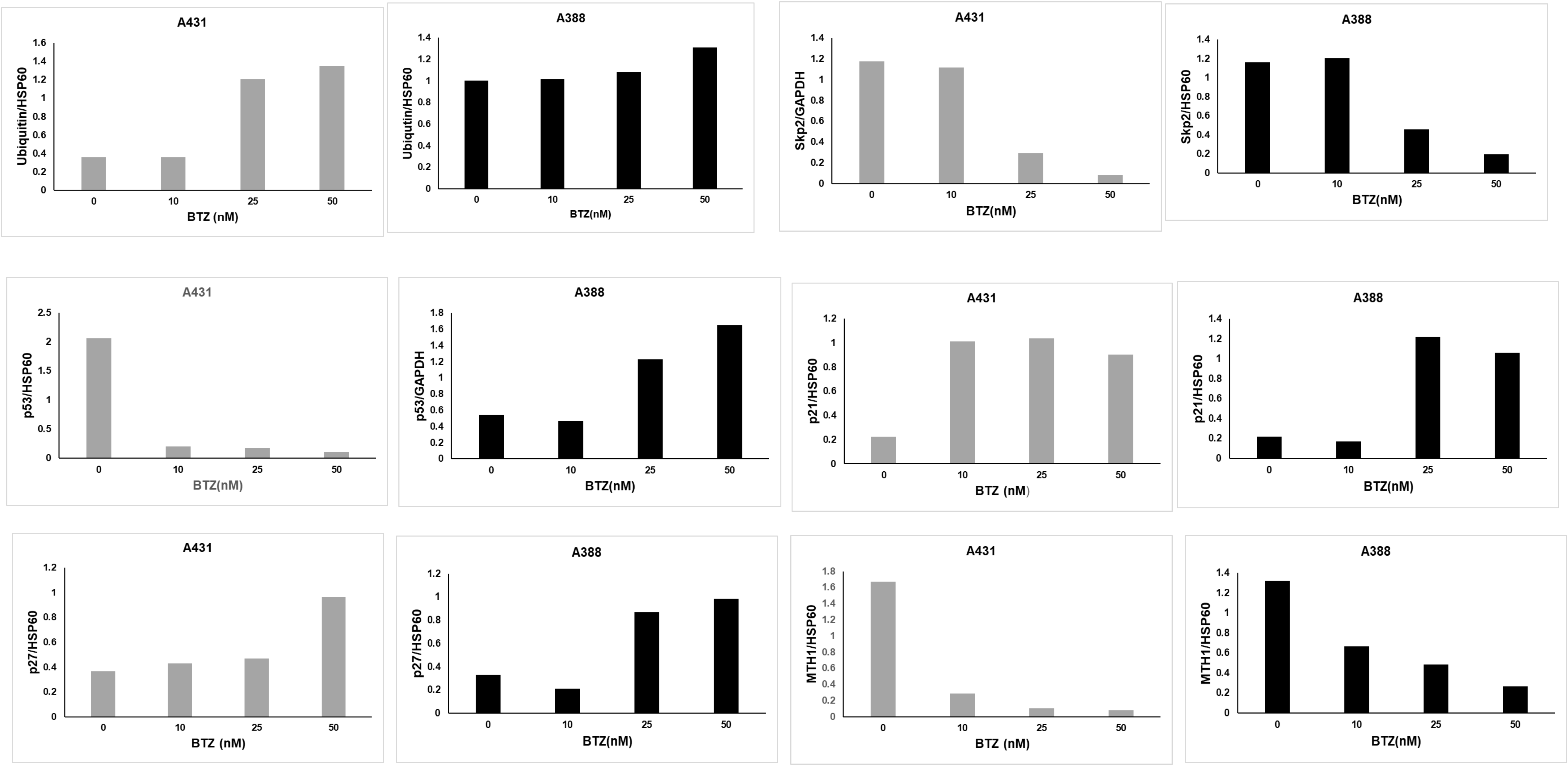
A431
Ubiquitin/HSP60
BTZ (nM)
A388
Ubiqutin/HSP60
BTZ(nM)
A431
Skp2/GAPDH
BTZ(nM)
A388
Skp2/HSP60
BTZ(nM)
A431
p53/HSP60
BTZ(nM)
A388
p53/GAPDH
BTZ(nM)
A431
p21/HSP60
BTZ (nM)
A388
p21/HSP60
BTZ(nM)
A431
p27/HSP60
BTZ (nM)
A388
p27/HSP60
BTZ(nM)
A431
MTH1/HSP60
BTZ (nM)
A388
MTH1/HSP60
BTZ(nM)
0
10
25
50

Figure 4B

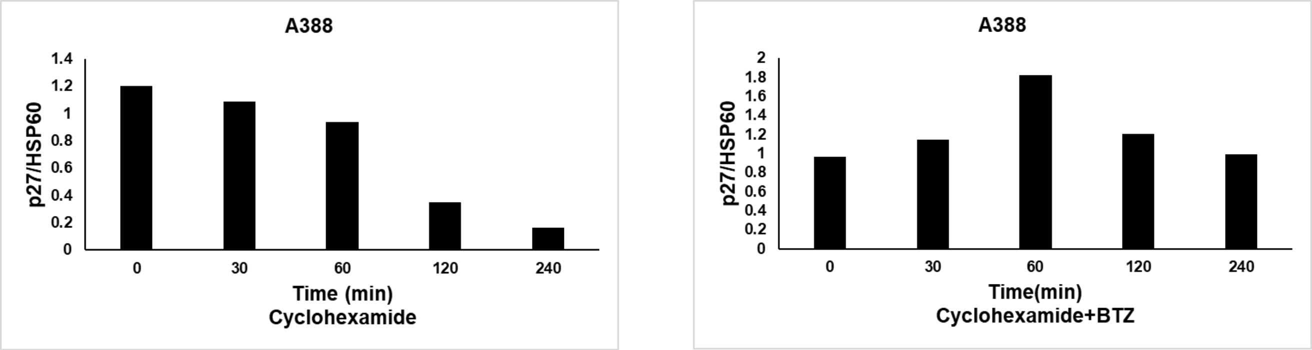

Figure 4C

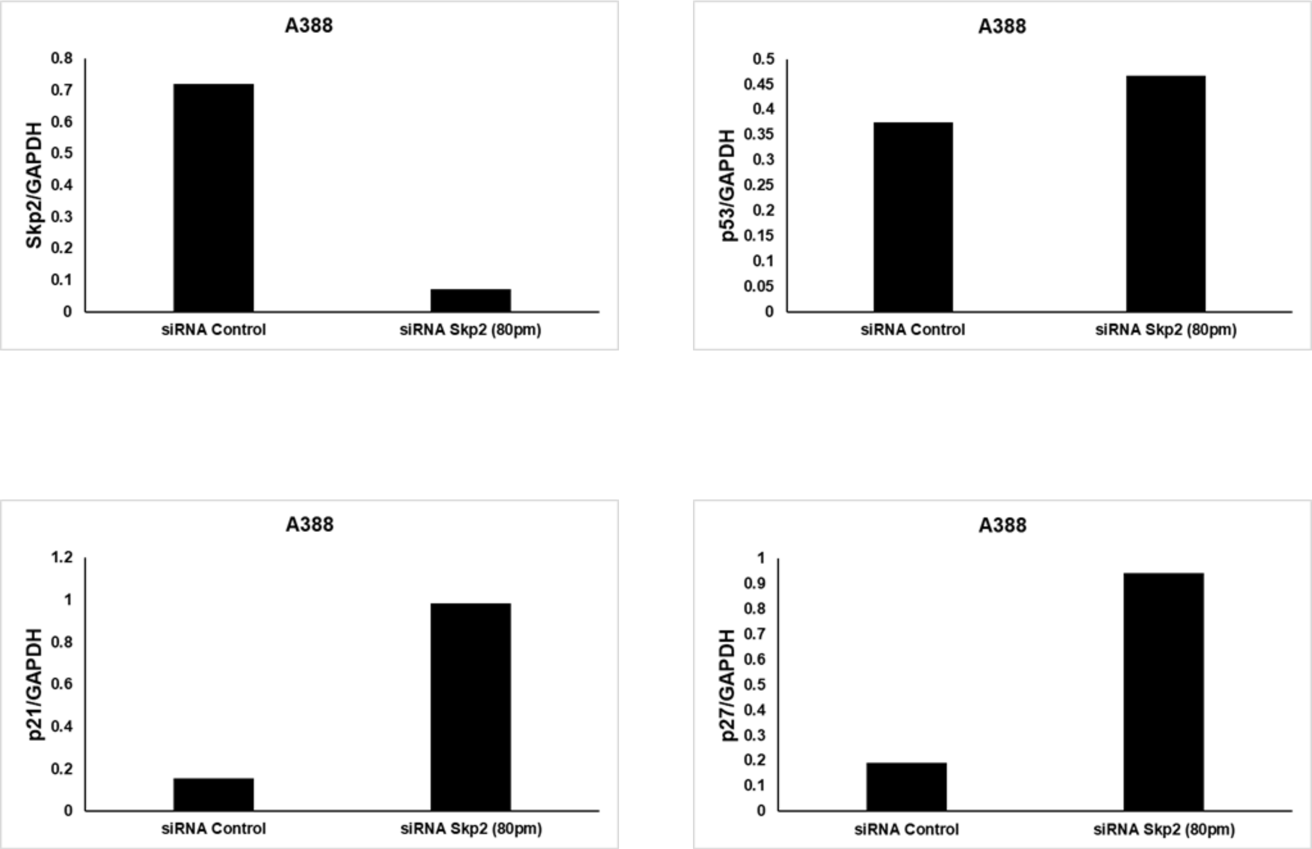

Figure 5A

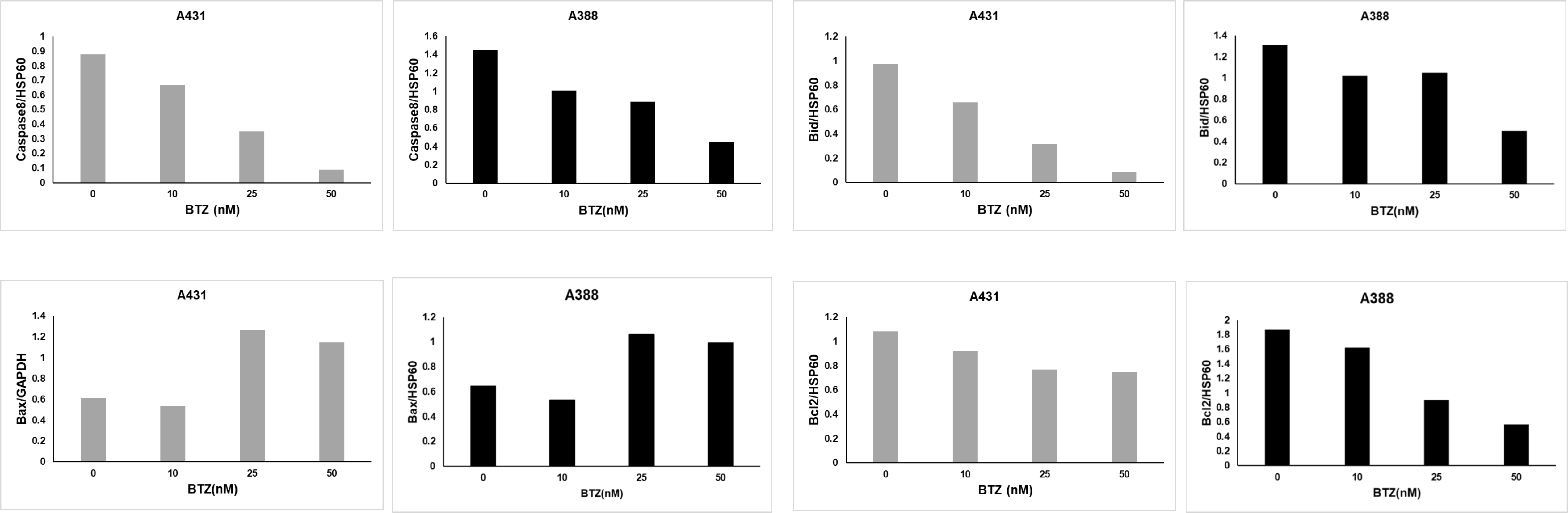

Figure5C

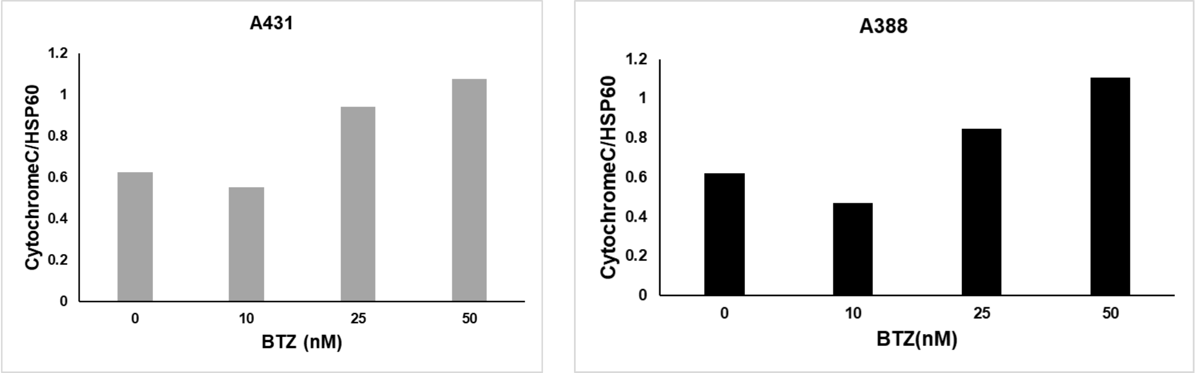

Figure 7D

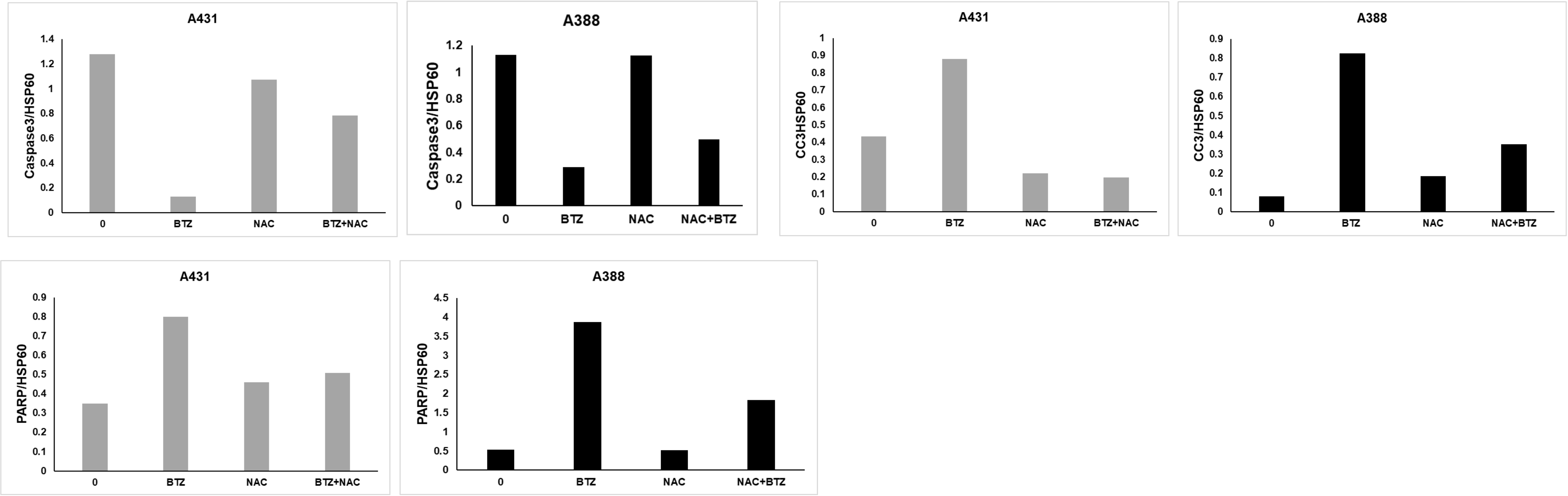

Supplementary Figure 3A

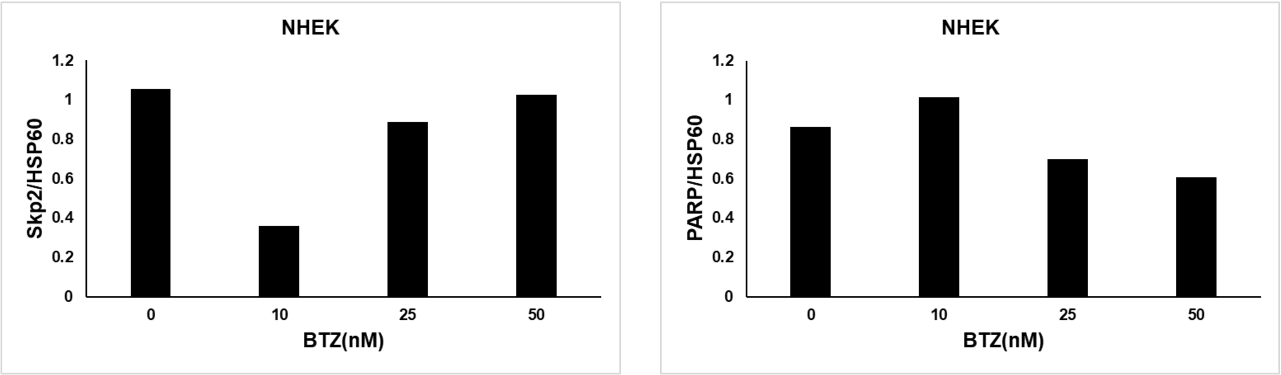

Supplementary Figure 3B

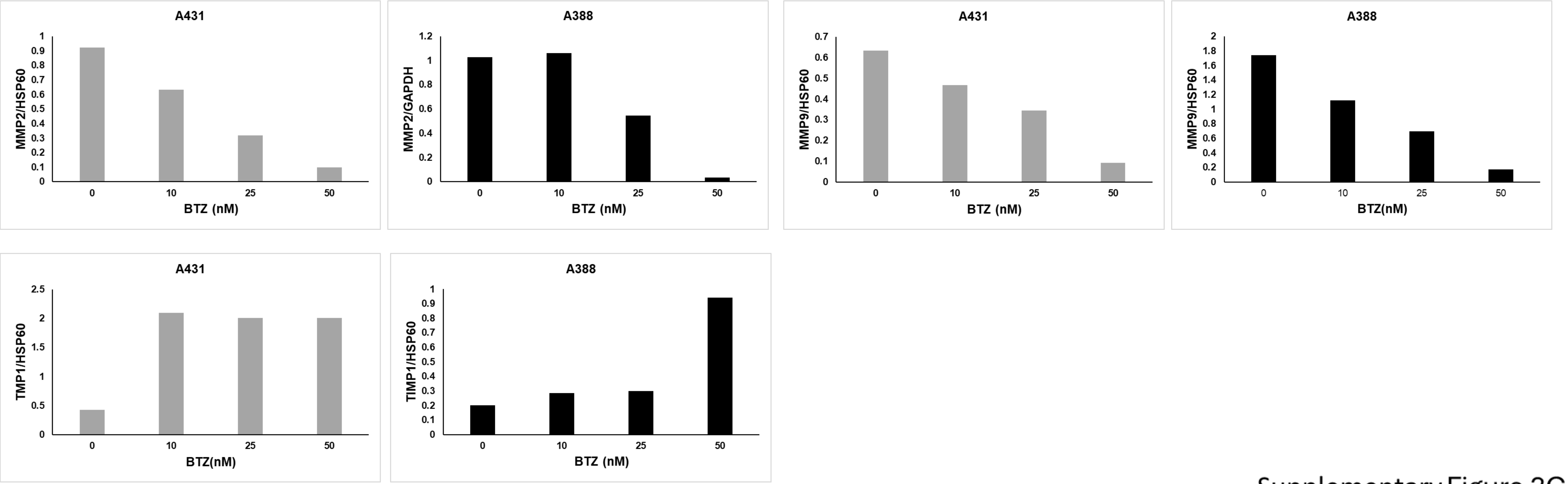

Supplementary Figure 3C

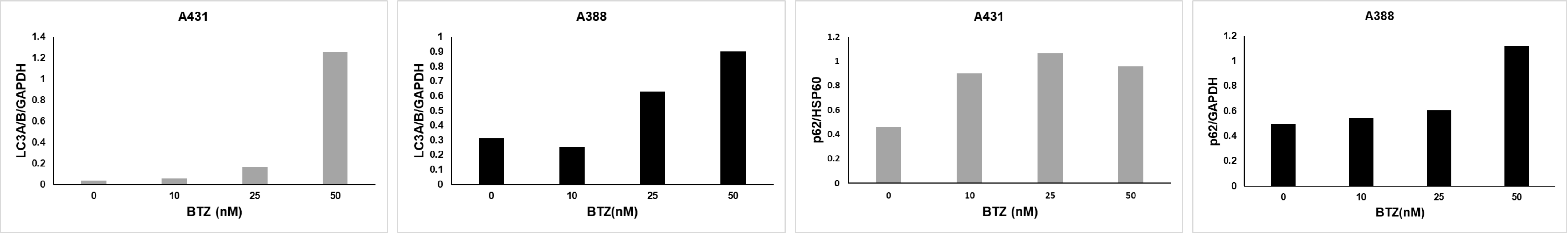

Supplement: Supplementary file 7 — Supplementary File 2 [file 41420_2024_1992_MOESM7_ESM.pdf]
